# Displaying R spatial statistics on Google dynamic maps with web applications created by Rwui

**DOI:** 10.1186/1476-072X-11-41

**Published:** 2012-09-24

**Authors:** Richard Newton, Andrew Deonarine, Lorenz Wernisch

**Affiliations:** 1 MRC Biostatistics Unit, Robinson Way, Cambridge, UK; 2 MRC Laboratory of Molecular Biology, Hills Road, Cambridge, UK

## Abstract

**Background:**

The R project includes a large variety of packages designed for spatial statistics. Google dynamic maps provide web based access to global maps and satellite imagery. We describe a method for displaying directly the spatial output from an R script on to a Google dynamic map.

**Methods:**

This is achieved by creating a Java based web application which runs the R script and then displays the results on the dynamic map. In order to make this method easy to implement by those unfamiliar with programming Java based web applications, we have added the method to the options available in the R Web User Interface (Rwui) application. Rwui is an established web application for *creating* web applications for running R scripts. A feature of Rwui is that all the code for the web application being created is generated automatically so that someone with no knowledge of web programming can make a fully functional web application for running an R script in a matter of minutes.

**Results:**

Rwui can now be used to create web applications that will display the results from an R script on a Google dynamic map. Results may be displayed as discrete markers and/or as continuous overlays. In addition, users of the web application may select regions of interest on the dynamic map with mouse clicks and the coordinates of the region of interest will automatically be made available for use by the R script.

**Conclusions:**

This method of displaying R output on dynamic maps is designed to be of use in a number of areas. Firstly it allows statisticians, working in R and developing methods in spatial statistics, to easily visualise the results of applying their methods to real world data. Secondly, it allows researchers who are using R to study health geographics data, to display their results directly onto dynamic maps. Thirdly, by creating a web application for running an R script, a statistician can enable users entirely unfamiliar with R to run R coded statistical analyses of health geographics data. Fourthly, we envisage an educational role for such applications.

## Introduction

Spatial statistical analysis with graphical display of data on geographic maps plays a core role in health geographics. Much of the pioneering work being done in the field of spatial statistical methodology is made available in packages from the R Project [1,2]. Google dynamic maps are a versatile source of global maps and satellite imagery, readily accessible via the internet [3]. We describe an easy to implement method for joining together these two freely available technologies.

Spatial statistics are used primarily in two related roles in health geographics, for research into spatial aspects of a disease and for disease prediction with a view to most accurately targeting health resources [4]. An example would be the application of remote sensing and geostatistical models to schistosomiasis control in Africa [5-8]. Google maps are already used in health geographics for surveillance and to aid disease management programs [9-11]. But to date, statistical analyses have not been directly integrated with the use of Google dynamic maps for data visualisation.

The R project contains many packages relevant to statistical analysis as applied to health geographics. All the data operations commonly required by a geographic information system are provided by packages such as sp [12], raster [13] and plotKML [14], and those packages that enable access through R, to projects such as SAGA [15] (rsaga [16]), GDAL [17] (rgdal [18]), GEOS [19] (rgeos [20]) and PROJ.4 [21] (proj4 [22]). Packages such as spatial [23], spatstat [24], gstat [25], DCluster [26] and others provide a huge variety of functions for performing spatial statistics. The Cran Task View ‘Spatial’ provides a useful summary of the packages available [27].

Graphical display of data on geographic maps is an important part of statistical analysis in health geographics. Previously we have described a method of displaying results from R scripts on Google static maps [28]. Google dynamic maps [3] have more ways of displaying data on a map and also allow the user to zoom and pan the map, so that the map, and any superimposed data, can easily be viewed at a huge range of spatial resolutions. They also allow the user to switch between different views, namely road, satellite or terrain maps. Because of this we have developed a method by which the results from an R script can be displayed directly onto a Google dynamic map.

**Figure 1:**
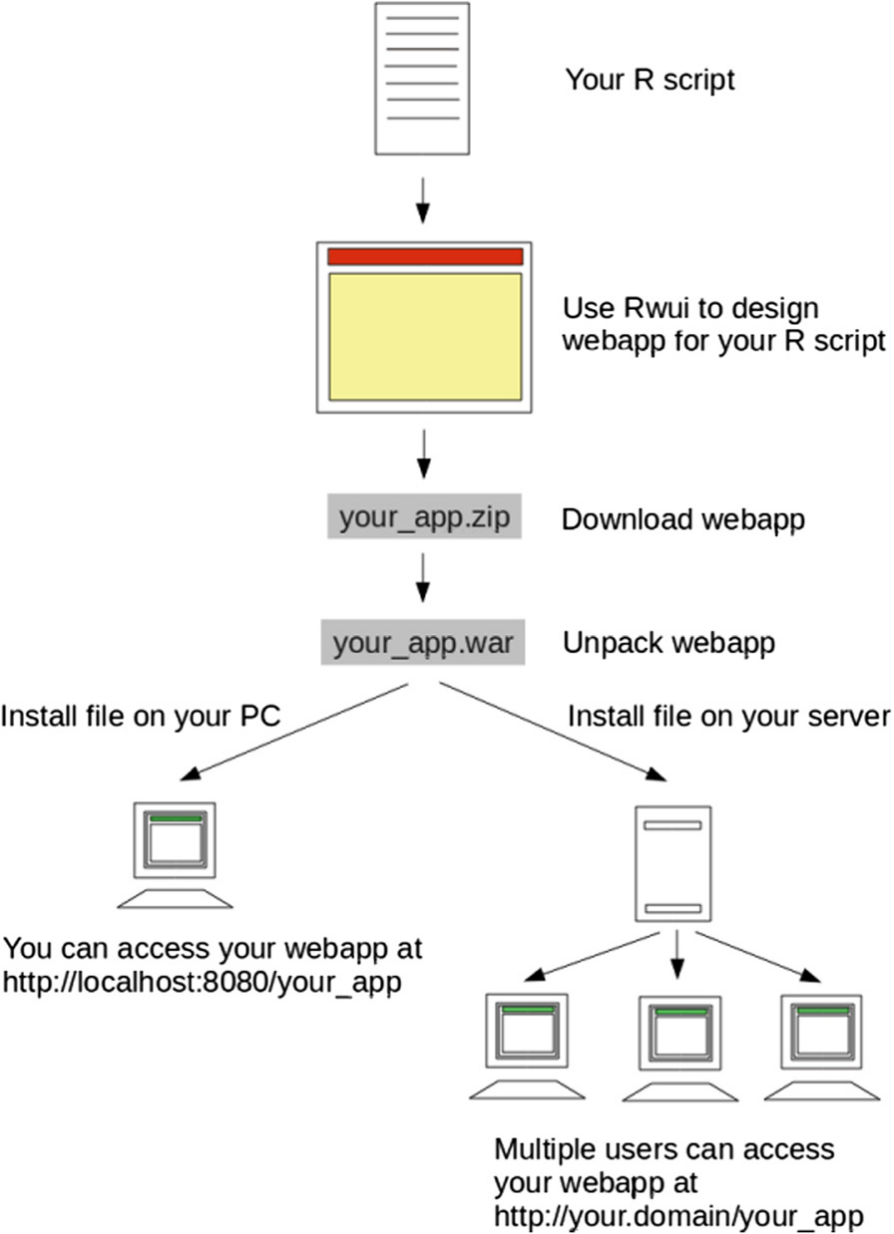
**Schematic flow diagram of procedure.** Showing how to create a web application using Rwui.

We achieve this by creating a Java based web application which runs the R script and then displays the results on the Google dynamic map. We provide technical details of exactly how this is done in Additional file 1. However we recognised that creating a Java based web application to run an R script would be time-consuming for anyone unversed in web programming, and this would limit the use of the method. Therefore in order to make the method easy for anyone to implement, even if they are entirely unfamiliar with creating Java based web applications, we have added the method to Rwui. Rwui is an established web application for *creating* web applications for running R scripts. A feature of Rwui is that all the code for the web application being created is generated automatically so that someone with no knowledge of web programming can make a fully functional web application for running an R script in a matter of minutes. Figure 1 shows a schematic flow diagram of the procedure.

Rwui allows a statistician, for example, to design a web application suitable for their R script on a sequence of web pages. By selecting options on Rwui’s web pages, a web application can be generated with no additional programming. Once Rwui has generated the web application, the person creating the web application downloads it from the final web page of Rwui. Once downloaded, the web application then needs to be installed on a suitable server in order to be used. Installing a web application created by Rwui is simply a matter of copying one file to the server. Full instructions are given in Rwui’s help file [29].

A typical web application created by Rwui presents users with a web page in their browser window in which they can upload data and/or select options such as parameter values. The web application runs the R script automatically, making the data and parameter values entered by the user available to the R script. On completion of the R script the results are returned to the user’s web page. Results can be displayed in a number of formats, selected when the web application is being designed, including the option of displaying results superimposed on a Google dynamic map. Results may be displayed on the map as discrete markers and/or as a continuous overlay. In addition, users of the web application may select regions of interest on the dynamic map with mouse clicks and the coordinates of the region of interest will automatically be made available for use by the R script at the next press of the ‘Analyse’ button.

Because the R script is run by a web application the analysis can, of course, be accessed remotely over the web, and by multiple users at any one time. It is however also possible to install the web application on a single stand-alone machine.

Integrating R scripts with Google dynamic maps in this direct manner has a number of potential uses. Firstly it allows statisticians, working in R and developing methods in spatial statistics, to easily visualise the results of applying their methods to real world data. Secondly, it allows researchers who are using R to study health geographics data for outbreak management, public health planning, and other geographic-data intensive tasks to display their results directly onto dynamic maps. Thirdly, by creating a web application for running an R script, a statistician for example, can enable users who are entirely unfamiliar with R to run the statistician’s R coded analysis of health geographics data. Users of web applications created by Rwui have no contact with the actual R script, which runs out of sight on the server; all that users see is the web pages of the application in their browser window. Fourthly, we envisage an educational role for such applications. By leveraging the interactive features of Google dynamic maps, various aspects of spatial statistical analyses can be explored in a visual, intuitive way.

**Figure 2:**
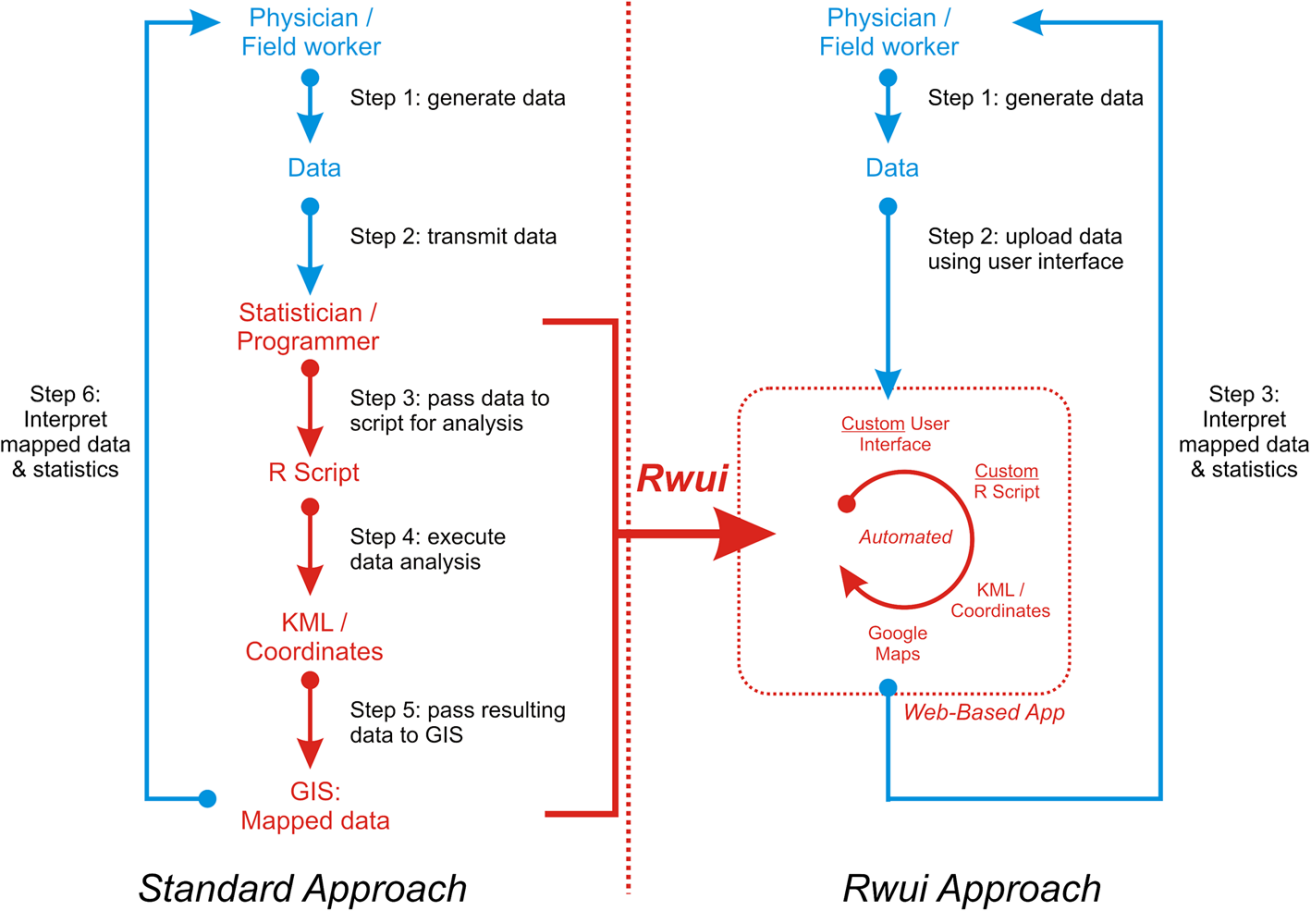
**Different approaches to the custom statistical analysis of health data, and integration with GIS visualization.** The standard approach, which involves using a statistical package in conjunction with a GIS system, is laborious and labour intensive and requires 6 steps shown here, which may preclude its usage in a time-sensitive, critical scenario such as an outbreak. The Rwui approach only requires 3 steps for repeated statistical analysis. The resulting webapp is platform independent and can be used on various devices (smartphones, PDAs, etc). And it is built around a custom R script, rather than being based around a limited number of built-in statistical analyses (as in EpiInfo and other related platforms).

Certain professions, such as field epidemiology and public health, require platforms that are capable of storing, processing and analyzing data using advanced, custom statistical routines, and facilitate the interpretation of these results through geospatial visualization. These tasks are commonly performed during a community health assessment, or the management of an outbreak. Hence, computational systems that can perform all of these tasks are essential [30]. Many clinicians performing this work do not have the skills or time to write custom statistical routines to process their data, then develop a user-friendly application (which could be used by other clinicians for data processing, rather than re-running complex scripts), and then import these results into a geospatial mapping platform for visualization. They will therefore need to send their data to a statistician for analysis. Statisticians will tend to use a combination of programs, such as a statistical language and a GIS platform, to analyze data [31]. This combination of statistical language and GIS requires expertise in different software packages, programming languages, and data formats. And usually, developing such a workflow will require a considerable amount of time, which may not be available during an outbreak or other crisis. The rapid application development provided by Rwui, which facilitates the integration of user friendly interfaces, statistical analyses, and GIS visualizations, addresses these difficulties. In addition, web-based distribution of the applications created by Rwui allows them to be easily accessed in the field using PDAs, smart phones, tablets, and other devices which are increasingly used in field epidemiology [30]. The flowchart on the left-hand side of Figure 2 illustrates a typical workflow where clinicians rely on statisticians to carry out a custom data analysis. The flowchart on the right-hand side of the figure illustrates a workflow using Rwui to create a web application.

There are a large number of technologies whose capabilities overlap those described here. None however provide the same combination of four key capabilities, 1. web accessible R statistical analysis combined with 2. the results overlain on dynamic map and satellite imagery and 3. the capacity for statisticians to create such web applications automatically without having to learn complex new programming skills and 4. the ability of users, who are unfamiliar with R programming, such as physicians and medical support staff, to execute data analyses in a user-friendly and efficient manner. A comprehensive review of all the overlapping technologies is not possible here, but some key ones will be summarized (see also feature matrix in Table 1). QuantumGIS [32] is a geographic information system (GIS) enabling the display and analysis of data but without the in depth spatial statistical analysis provided by R or the capabilities of web access. Mapserver [33] allows data to be presented on web accessible maps but again lacks the spatial statistical analysis provided by R. SOLVAT [34] provides a method for analyzing data using OLAP (online analytical processing) but is a Windows based executable, and uses its own built-in mapping system that cannot be shared. The EpiInfo [35], a program developed for Windows, allows mapping using a built-in mapping system, and some statistical functions, but not a full statistical language, or web-based maps. With ArcGIS [36] customised GIS web applications can be created but again without R, and ArcGIS is not open source or free.

**Table 1:** Comparing the features provided by Rwui with those of other systems

**System**	**Description**^**1**^	**Free**^**2**^	**Open source**^**3**^	**Visualization method**^**4**^	**User interface**^**5**^	**Automated visualization**^**6**^	**Spatial Statistics**^**7**^	**Compatible with R**^**8**^	**Gui created**^**9**^
QuantumGIS	Executable Program	Yes	Yes	Various	Yes	Yes	Some	No	No
Mapserver	Web server	Yes	Yes	Various	Yes	Yes	No	No	No
ArcGIS	Executable Program	No	No	ESRI	Yes	Yes	Yes	No	Yes
Epi Info	Executable Program	Yes	No	ESRI	Yes	Yes	Some	No	No
SOLVAT	Executable Program	Yes	Yes	ESRI	Yes	Yes	Some	No	No
plotKML	R package	Yes	Yes	Google	No	Yes	Yes	Yes	No
plotGoogleMaps	R package	Yes	Yes	Google	No	Yes	Yes	Yes	No
googleVis	R package	Yes	Yes	Google	No	Yes	Yes	Yes	Yes
Rwui	Webapp creating webapps	Yes	Yes	Google	Yes	Yes	Yes	Yes	Yes

^1^Type of software system.

^2^Is the software free or commercial.

^3^Is the system’s source code available for use.

^4^The type of visualization system used.

^5^Is a graphical user interface available.

^6^Are maps automatically produced, or is further programming required?

^7^Can advanced spatial statistics be incorporate and customized?

^8^Is the software system able to incorporate R code?

^9^Can a custom, easy to use GUI driven stand-alone application be created?

plotKML [14] is a useful R package that writes R spatial classes to KML (Keyhole Markup Language) files and outputs these for display in Google Earth [37]. plotGoogleMaps [38] is another R package for plotting data on Google dynamic maps. googleVis [39] is a sophisticated R package that allows a user to visualise R data frames using the Google Visualisation API [40] which includes access to dynamic maps. All three packages are excellent tools for a statistician who wishes to view or publish the results of their R analysis of their own data on dynamic maps. However these packages are not designed to fulfil the primary role provided by Rwui. Webapps created by Rwui enable the statistician to provide remote access to their R script to users entirely incognizant of R so that those users can analyse their own data, with the statistician’s R script, and view the results on Google maps, without even knowing they are using R. It would certainly be possible to wrap further web code around plotKML and plotGoogleMaps packages to provide this functionality, but this would require an in depth knowledge of web programming. googleVis provides details as to how to embed googleVis in websites but a certain amount of knowledge is currently still required to merge the necessary technologies to create a fully functional remotely accessible webapp. The purpose of Rwui is to relieve the statistician from having to learn new technologies by automating the process of creating a webapp. There are alternative ways of creating web interfaces for R scripts, details of which can be found in the R-FAQ on R web interfaces [41], which require varying amounts of web programming skills and currently lack the facility of returning results to Google dynamic maps. Rwui fills a niche where a statistician requires both R statistical analysis and display of data on dynamic maps for users ignorant of R in a manner which obviates the statistician from learning any of the underlying technology.

In the following sections we first explain the method and then present two example applications where results from an R analysis are displayed on a Google dynamic map.

## Methods

### Running an R script from a web application

By using Rwui, a web application for running an R script can be created without having to know any technical details and without having to do any web programming, but in this section we give a brief summary of the underlying methods.

In order to run an R script from a web application we use a Java based approach. The web application has three main components; the View (the way in which information is presented to the user), the Controller (controlling the flow of the application) and the Model (the data processing). The Model part of the application, a Java program, passes the information entered by the user on the application’s web page to the R script and then runs the script. The application waits for the R script to finish and then returns the results to the web page.

The R script is run using R batch mode. The batch command is placed in a shell script, which is run as a Process using the Java application’s instance of the Runtime class. Before the R script is run, the values of the variables that the user entered on the web pages are passed to the R script. The application writes this information, as R assignments, into a text file which is concatenated with the main R script prior to execution. The application waits for the script to finish and then displays the results on the web page. Google dynamic maps are generated by Javascript, so in order to display results on a Google dynamic map the results, written by the R script, need to be in the form of an appropriate Javascript file.

In Additional file 1 we elaborate on the technical details as to how an R script can be run by a Java based web application. But by using Rwui, such a web application can be created without having to know any of these technical details. In the following section we describe how to use Rwui. We then describe how to install an application once it has been created by Rwui. Then we explain the R script requirements for displaying results on a Google dynamic map, namely the Javascript file that the R script needs to produce. In the final Methods section we describe the use of a typical application created by Rwui that displays results on a Google dynamic map.

### Creating a web application for running an R script using Rwui

Rwui is a web application, accessible in a web browser at the following address: http://sysbio.mrc-bsu.cam.ac.uk/Rwui. Anyone with an R script can use Rwui to design and create a web application for running their R script. Rwui comprises a sequence of twenty web pages on which the user designs their web application, tailoring it to the requirements of their R script. The user can backtrack, edit and insert features. A facsimile of the web application they are creating is displayed in the lower part of the current Rwui web page as the user’s design of their web application progresses.

**Figure 3:**
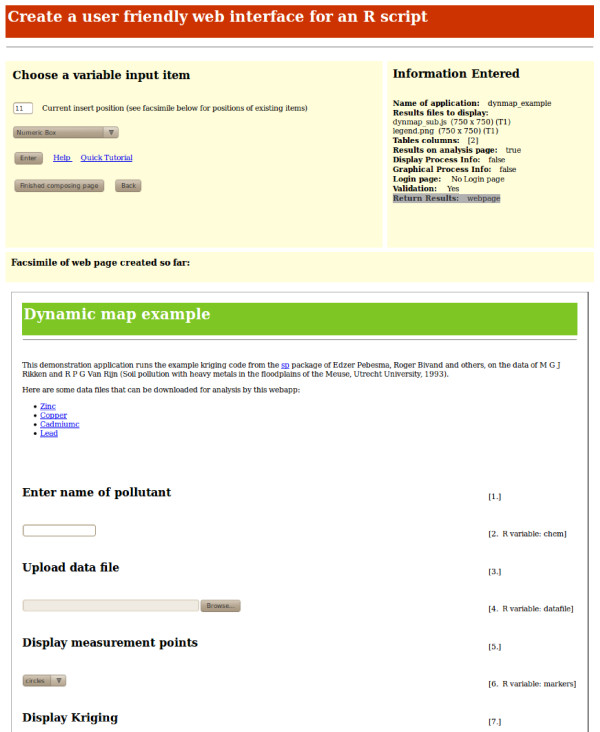
**One web page of Rwui, on which input items are added.** One of the twenty web pages that comprise Rwui. A facsimile of the dynamic map example application being created can be seen on the lower half of the page.

After entering a title and introductory text for the application, the user of Rwui has to select the input items that will appear on their application’s web page. Input items may be Numeric entry boxes, Text entry boxes, Text Area boxes, Radio Buttons, Checkboxes, Drop-down lists, Multi-select lists, File Upload boxes, Replicate File Upload entry, Zip File Upload boxes. Each of the input variables of the R script, that is, those variables in the script that require a value supplied by the user, must have a corresponding input item on the application’s web page. In a completed web application the association of an input item on the web page with an input variable in the R script works in the following manner. If, for example, the R script variable my_num is associated with a Numeric entry box, then the completed application will automatically add a line to the beginning of the R script that assigns to the R variable my_num whatever value the user of the completed application enters in the Numeric entry box on the web page. For example, if the user of the completed application enters 1.234 in the Numeric entry box then the line my_num <- 1.234 will be written automatically at the beginning of the R script before the script is run. Figure 3 shows a web page from Rwui. On this page the input items that will appear in the application are added; the facsimile of the web application that is being created can be seen on the lower half of the page.

The names of the results files to be displayed for the user of the completed web application also need to be entered whilst the application is being designed in Rwui. Specifying that the results are to be displayed on a Google dynamic map is simply a matter of checking a box and giving the name of the javascript file that the R script will write containing the code for creating the map. This javascript file is explained in the section ‘R script requirements to display results on a Google dynamic map’. There is also the option of displaying the results on a *clickable* Google dynamic map. If this option is chosen, in the completed web application mouse clicks can be used to mark out a region of interest (ROI) on the dynamic map. ROI’s may be circles, squares, rectangles or irregular polygons and the coordinates of the ROI are automatically made available to the R script. Further information on ROI’s can be found in the ‘Example applications’ section.

Subsequent pages of Rwui allow the user to specify the layout of results and add a number of optional features if required. For example the application can optionally include a login page with access controlled either by a single password, or by username/password pairs. Then once the user of Rwui has finished designing their web application Rwui generates all the code for the web application automatically. The completed web application can be downloaded from the final web page of Rwui.

### Installing applications created by Rwui

The download contains a compiled, ready-to-use copy of the web application as a .war file which needs to be installed on a suitable server. Web applications created by Rwui run on Tomcat servers [42]. Tomcat is free server software (under Apache License, Version 2.0 [43]), easy to install on both Unix and Windows machines, and with an impeccable security record. Installing a web application created by Rwui is simply a matter of copying the .war file to the server’s Tomcat webapps directory. Tomcat itself requires Java [44] installed. Full instructions for installing Tomcat and Java, and for installing web applications created by Rwui, are given in Rwui’s help file [29].

Most users will not need to modify the source code of the application but, for the benefit of anyone who would like to make modifications of their own to the application the download also contains all the source code and an Ant [45] script for rebuilding the application. In addition the download contains an XML application description file. This summarises all the information about the web application in a form Rwui can read, so if a user needs to redo an application at a later date they simply have to upload the application description file into Rwui.

Tomcat and the web applications created by Rwui can also be installed on individual stand-alone machines, in which case the web applications are accessed in a browser on the machine via the localhost address [29]. This facility is useful when developing applications prior to installing on a remote server for general access by multiple users. However we envisage it will also be useful for researchers performing a spatial statistical analysis of data with their own R script who want to view the results of their analysis on a Google dynamic map. By using the localhost address they can do this on a stand-alone machine and don’t have to install the web application on a remote server. The stand-alone machine does need to be internet connected in order to access the Google dynamic maps.

### R script requirements to display results on a Google dynamic map

Google dynamic maps are actually created by javascript code, from the Google Maps JavaScript API v3 [46], placed on the web page. The R script associated with a web application that is designed to display results on a Google dynamic map must write this javascript code to a file. The web application will then automatically include this file of javascript at the correct place in the web page. Here is an example of javascript code that, when written by the R script to a file, will display a Google dynamic map on the web page:

function initialize() {

var latlng = new google.maps.LatLng(50.975, 5.74);

var myOptions = {

zoom: 8,

center: latlng,

mapTypeId: google.maps.MapTypeId.ROADMAP

};

var map = new google.maps.Map(

document.getElementById(‘map_canvas’),

}

**Figure 4:**
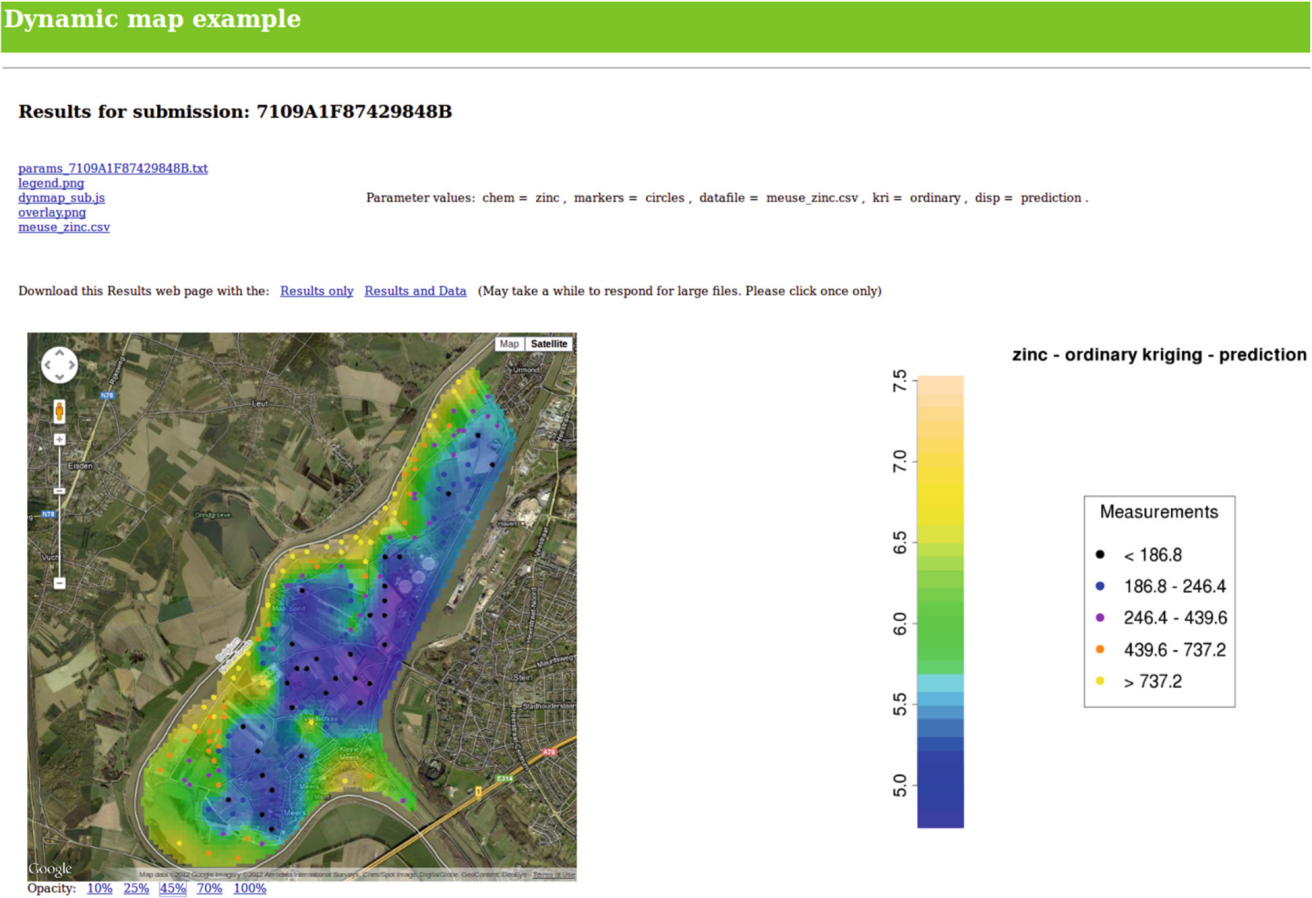
**Meuse soil pollution example application.** Screenshot of part of the web page of the Meuse soil pollution example application http://sysbio.mrc-bsu.cam.ac.uk/dynmap_example. Individual data points are marked as colour-coded circles and the results of kriging displayed as an overlay.

This code produces a basic map, a roadmap centred at Latitude 50.975, Longitude 5.74 and with zoom factor 8. This is actually the map in Figure 4, but without the data that Figure 4 overlays on the map. Further javascript code is required to add data to the map. Data can be included on a map in the form of markers, circles, polygons, polylines and overlays. The javascript code for adding these items is simple, and well documented in the Google Maps Javascript API [46]. But in order to help write the necessary javascript we have created R functions which produce the correct javascript for displaying data on a dynamic map. The R functions are available from Rwui’s documentation on adding dynamic maps [47] and are included here as Additional file 2 and documented in Additional file 3. Given a data frame of coordinates and the map options (latitude/longitude of centre, initial zoom factor etc) the R functions will write the javascript file needed to display the data on the required dynamic map as standard markers, custom markers or circles of varying radii and/or colour.

As well as discrete markers, a continuous overlay can also be displayed on a Google dynamic map, that is, a png image which is displayed on top of the map with a variable degree of transparency so that details of the underlying map can still be seen. Normally, for Google dynamic maps to display an overlay the png file has to be located on a publicly accessible server. This presents problems for applications created by Rwui since the architecture of these applications holds all results files in directories below the WEB-INF directory where they are never publicly accessible. It also presents problems for anyone using a web application on a stand-alone machine, using the localhost address, rather than on a server, since in general no files on such a machine will be publicly accessible.

The solution is to use the ProjectedOverlay javascript class [48,49]. This file needs to be downloaded [50] and copied to the top level directory of an installed web application. With this file present a png file written by the application’s R script, and designated as being an overlay in the javascript code, will be overlain on the dynamic map. The R helper functions (Additional file 2) can be used to write the javascript code necessary for designating the png file as an overlay, simply requiring the name of the png file, and the latitude and longitude of the overlay’s south-west and north-east corners. Further details can be found in Rwui’s documentation on adding dynamic maps [47], which is included here as Additional file 4.

Depending on how an overlay is produced it may need to be converted to the projection used by Google Maps. Simply setting the SW and NE lat/longs of the overlay is not sufficient because, if the overlay was generated in a different projection system, it will be warped in the Google Maps projection. The R package raster [13] has simple to use functions for changing a raster of values from one projection system to another. This uses the R package rgdal [18], which requires the PROJ.4 library [21] and GDAL [17] to be installed.

There are examples of the use of the raster package for converting projections to create overlays for Google dynamic maps in the R code of the example applications (see Additional files 5, 6 and 7).

Data in the form of KML (Keyhole Markup Language) files can be displayed directly on to Google dynamic maps. The R package plotKML [14] provides a useful suite of functions for writing data in the form of a number of different R spatial classes to KML files. As with overlays, for Google dynamic maps to display a KML file, normally the file has to be located on a publicly accessible server, which presents problems for applications created by Rwui. The solution is to use the geoxml3 KML processor [51]. This file needs to be downloaded [52] and copied to the top level directory of an installed web application. With this file present a .kml file written by the application’s R script, and designated as being a KML file in the javascript code, will be added to the dynamic map. The R helper functions (Additional file 2) can be used to write the javascript code necessary for designating the file as a KML file, simply requiring the name of the .kml file. Further details can be found in Rwui’s documentation on dynamic maps [40] (Additional file 4).

There are step-by-step guides to creating two example web applications that display results on a Google dynamic map [53] which are included here as Additional file 8. Table 2 gives a summary of all the documentation and resources available for Rwui and the external resources that are required.

**Table 2:** List of Rwui resources and documentation, required external resources and useful external resources

	
**Rwui resources and documentation**	
Rwui	http://sysbio.mrc-bsu.cam.ac.uk/Rwui
Documentation: Rwui	http://sysbio.mrc-bsu.cam.ac.uk/Rwui/tutorial/Instructions.html
Step-by-step guide: Basic webapp	http://sysbio.mrc-bsu.cam.ac.uk/Rwui/tutorial/quick_tour.html
Documentation: Displaying dynamic maps	http://sysbio.mrc-bsu.cam.ac.uk/Rwui/tutorial/dynamic_map_tutorial/dynmap_docs/index.html
Step-by-step guides: Dynamic map webapp	http://sysbio.mrc-bsu.cam.ac.uk/Rwui/tutorial/dynamic_map_tutorial/dynmap_step_by_step/index.html
R functions: creating dynamic map javascript	http://sysbio.mrc-bsu.cam.ac.uk/Rwui/tutorial/dynamic_map_tutorial/dynmap_functions.R
Documentation: R functions	http://sysbio.mrc-bsu.cam.ac.uk/Rwui/tutorial/dynamic_map_tutorial/dynmap_functions_docs/index.html
Example app: Simple	http://sysbio.mrc-bsu.cam.ac.uk/dynmap_simple_example
R code: Simple app	http://sysbio.mrc-bsu.cam.ac.uk/Rwui/tutorial/dynamic_map_tutorial/dynmap_simple_example.R
Example app: Meuse	http://sysbio.mrc-bsu.cam.ac.uk/dynmap_example
R code: Meuse app	http://sysbio.mrc-bsu.cam.ac.uk/Rwui/tutorial/dynamic_map_tutorial/dynmap_example.R
Example app: Malawi	http://sysbio.mrc-bsu.cam.ac.uk/click_dynmap_example
R code: Malawi app	http://sysbio.mrc-bsu.cam.ac.uk/Rwui/tutorial/dynamic_map_tutorial/click_dynmap_example.R
R code: preprocessing population data	http://sysbio.mrc-bsu.cam.ac.uk/Rwui/tutorial/dynamic_map_tutorial/preprocess.R
**External resources required**	
Tomcat	http://tomcat.apache.org/
Java	http://www.oracle.com/technetwork/java/javase/downloads/index.html
Google Maps API key	https://developers.google.com/maps/documentation/javascript/tutorial#api_key
ProjectedOverlay	http://code.google.com/p/geoxml3/source/browse/trunk/ProjectedOverlay.js
**Useful external resources for managing projections**	
R packages: raster, sp, rgdal, geosphere, rsaga, proj4, rgeos, plotKML	http://cran.r-project.org/web/packages/
GDAL	http://www.gdal.org/
PROJ.4	http://trac.osgeo.org/proj/
GEOS	http://trac.osgeo.org/geos/
SAGA	http://www.saga-gis.org/en/index.html

### Using a web application created by Rwui

A web application created by Rwui presents users with a web page in their browser window in which they can upload data and/or select options such as parameter values. Clicking the ‘Analyse’ button sends their data and parameter values to the server where the web application runs the R script. The data and parameter values entered by the user are automatically made available to the R script. On completion of the R script the results are returned to the user’s web page.

Prior to running the R script the application checks the validity of the values that the user has entered and returns an error message to the page if any are invalid. Only numeric values or NA can be entered into Numeric entry boxes. Numeric, Text and File Upload boxes cannot be left blank. When creating the application validation may be turned off, although this is not recommended.

On completion of the analysis, the results are displayed in the lower half of the web page. Below the results a link appears to a Results page for this particular submission (that is, for the last activation of the ‘Analyse’ button). The user can change data files and/or the values of any of the variables and/or the shape and/or size of the region of interest, and re-analyse, and the new results will appear on the web page and another link will be added at the bottom of the page, and so on. Clicking on a link brings up a dedicated Results page for the corresponding submission.

If an analysis will take some time to complete then, when designing the application with Rwui, it is possible to specify that real time progress information is displayed for the user whilst the application is running.

## Example applications

### Example 1: Meuse soil pollution

The first example application can be found at http://sysbio.mrc-bsu.cam.ac.uk/dynmap_example. This dem- onstration application runs the example kriging code from the R package sp [12], on the Meuse River soil pollution data [54]. Figure 4 shows a results page from this application. The individual pollution measurements can be marked on the map as either colour-coded or radius-coded circles. The output from the kriging is overlain on the map. Controls to select the transparency of the overlay are automatically included below the map. From the map in Figure 4, a strong trend can be observed, with high zinc levels being closest to the river. A few inland regions with elevated levels, which can be easily explored using the interactive map, also become immediately apparent. The R code that reads the data file, performs the kriging, creates the overlay file and writes the javascript file can be found in Additional file 5.

### Example 2: Malawi population density databases

**Figure 5:**
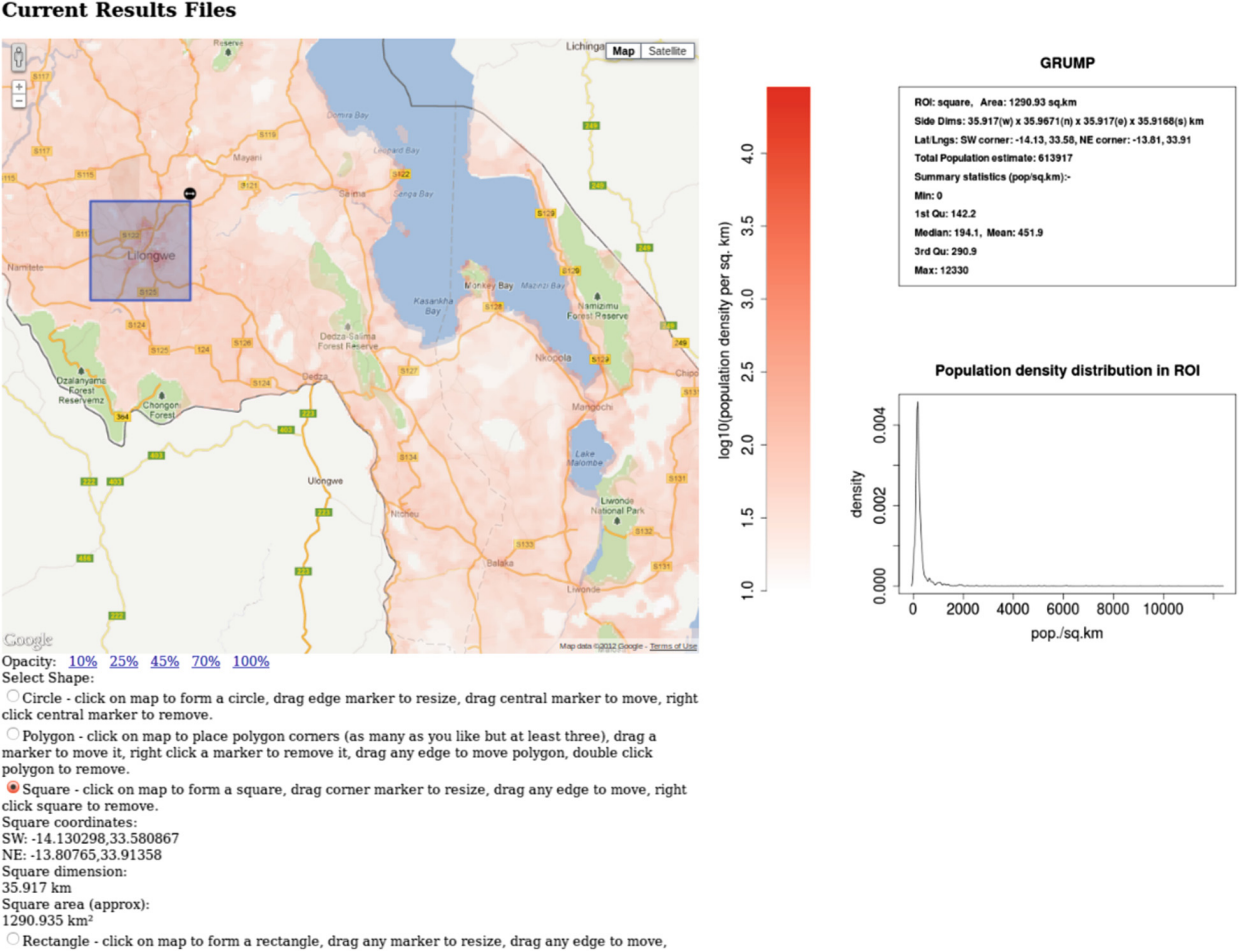
**Malawi population density example application.** Screenshot of part of the web page of the Malawi population density example application http://sysbio.mrc-bsu.cam.ac.uk/click_dynmap_example. Data from the GRUMP database is displayed. A square region of interest has been selected by the user, and the summary statistics for this area can be seen on the right of the screen.

**Figure 6:**
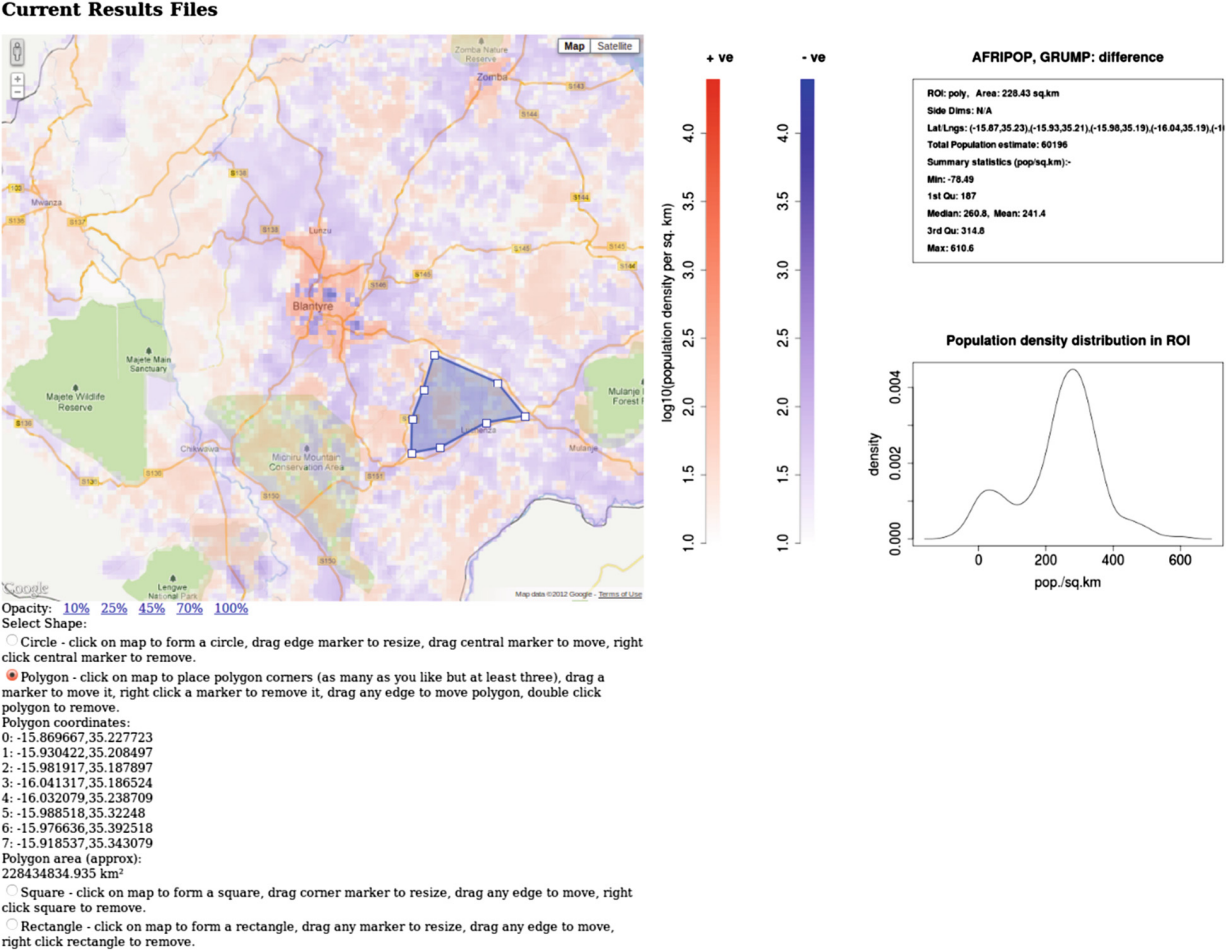
**Malawi population density example application.** Screenshot of part of the web page of the Malawi population density example application http://sysbio.mrc-bsu.cam.ac.uk/click_dynmap_example. The difference between the Afripop and GRUMP databases is displayed. An irregular polygon region of interest has been selected by the user, and the summary statistics for this area can be seen on the right of the screen.

The second example application, found at http://sysbio.mrc-bsu.cam.ac.uk/click_dynmap_example, demonstrates a clickable dynamic map. As well as the overlay transparency controls, radio buttons are automatically added below the map by which the type of region of interest (ROI) can be selected. ROI’s may be circles, squares, rectangles or irregular polygons. The ROI is added to the map by mouse clicks and its size and/or shape adjusted by mouse drags. On pressing the ‘Analyse’ button the latitude and longitude coordinates of the ROI are made available to the R script prior to it being run. This example application displays an overlay showing the population density of Malawi from three different population databases GRUMP [55], GPW [56] and Afripop [57]. Databases giving estimates of population densities are essential in disease prediction [58], and there are a number of databases available using slightly different methodologies for estimating population densities. The example application compares the population density information for the country of Malawi contained in three such databases. The data from a single database may be displayed, or the mean of two or three, or the difference of any two. If a ROI is drawn then pressing the ‘Analyse’ button will return summary statistics for the population density in the selected ROI. Figures 5 and 6 show results pages from this application. By rendering the data from the different databases into an interactive map, discrepancies between databases can be visually explored and interrogated easily by creating ROIs. Differences in data may indicate significant differences in methodologies and be of particular interest.

In this example the data displayed on the map is not uploaded by the user. The data from the three databases was preprocessed into a suitable form and saved as .Rdata files. These were bundled as subsidiary files with the application when it was created with Rwui. The R code used to preprocess the raw data from the three databases is given in Additional file 6. The R code that reads in the .Rdata files, creates the correct overlay and produces the summary statistics for the selected ROI can be found in Additional file 7.

## Conclusions

We have developed a free and easy to implement method by which statisticians working in R can display results on dynamic maps. Using this method statisticians can produce customised web accessible geographic information systems dedicated to specific spatial statistical analysis problems. We think this will be a useful additional tool in the range of free tools currently available in the field of health geographics. The web applications created by Rwui are open source, as is R, but users should note that Google dynamic maps are not open source.

There are a number of advantages of using a web interface to an R script. It means that the R analysis can be used by anyone even if they do not know any R, or even have R installed on their machine. The R analysis can be made available remotely via the internet, and updating the R script is simply a matter of updating the one copy on the server. The benefit to the statistician in using Rwui to create such a web application is that they do not need to learn java-based web programming in order to create a web interface for their R script, all the programming is done automatically. The disadvantage in using Rwui, rather than programming the whole application from the beginning, is inevitably some reduction in flexibility in the design of the application. Rwui has however been created to include most features commonly required by users. The benefits of Google dynamic maps are two-fold. Firstly the ability to swap between map and satellite imagery. Secondly the ability to zoom and pan so that maps can easily be examined at different levels of resolution. A limitation of Google dynamic maps is that they do require an internet connection. With a slow internet connection Google dynamic maps may take a while to load, especially with complex overlays. R itself can be relatively slow software, but including C++ with R code can improve speeds greatly. A current limitation of web applications created by Rwui that display results on Google dynamic maps is that only single regions of interest may be drawn. The applications are also currently one page applications. They are however open source so can provide a foundation for coding more complex applications containing a series of web pages.

Future work will concentrate on extending the capabilities of the method; for example allowing a user to create multiple regions of interest on a map. We also aim to produce further helper functions to convert the output from R packages into Google Maps API javascript code and to produce a downloadable open source version of Rwui.

## Competing interests

The authors declare that they have no competing interests.

## Authors’ contributions

RN coded Rwui and the example applications. All authors contributed to the concept and design of the work, read and approved the final manuscript.

## Supplementary Material

Additional file 1 Technical details - how to run an R script in a Java based web application. Click here for file

Additional file 2 R functions for writing Javascript for displaying Google dynamic maps with data overlays. Click here for file

Additional file 3 Documentation for R functions for writing Javascript for displaying Google dynamic maps with data overlays. Click here for file

Additional file 4 Documentation on using Rwui to create a web application displaying results on a Google dynamic map. Click here for file

Additional file 5 **R code for the Meuse soil pollution example application at**http://sysbio.mrc-bsu.cam.ac.uk/dynmap_example**.** Click here for file

Additional file 6 **R code for pre-processing database downloads for Malawi population density clickable example application at**http://sysbio.mrc-bsu.cam.ac.uk/click_dynmap_example**.** Click here for file

Additional file 7 **R code for the Malawi population density clickable example application at**http://sysbio.mrc-bsu.cam.ac.uk/click_dynmap_example**.** Click here for file

Additional file 8 Step-by-step guide to using Rwui to create webapps that display R script results on Google dynamic maps. Click here for file
