# Supplementary material for: Displaying R spatial statistics on Google dynamic maps with web applications created by Rwui
Source: Int J Health Geogr. 2012 Sep 24;11:41. doi: 10.1186/1476-072X-11-41 (PMC3548681; doi:10.1186/1476-072X-11-41)
Supplement: Additional file 1 — Technical details - how to run an R script in a Java based web application. [file 1476-072X-11-41-S1.pdf]

Additional File 1: Technical details - how to run an R script in a Java based web application

Richard Newton, Andrew Deonarine and Lorenz Wernisch

June 19, 2012

Introduction

The structure of web applications created by Rwui demonstrates how a Java based web application can be constructed so as to run an R script. The web applications use the Apache Struts framework [1]. Struts is an open source and popular Java based framework for constructing web applications. The Apache Struts framework separates the three main components of a web application; the View (the way in which information is presented to the user), the Controller (controlling the flow of the application) and the Model (the data processing). This produces a web application that is well-organised, stable and extensible. The Model part of the application, a Java program, passes the information entered by the user on the application's web page to the R script and then runs the script. The application waits for the script to finish and then displays the results on the web page. If the script takes some time to run, the web application can display progress information for the user.

The R script is run using R batch mode. The batch command is placed in a shell script, which is run as a Process using the application's instance of the Runtime class. The Process class has a method that causes the current thread to wait until the Process has completed. Before the R script is run, the values of the variables that the user entered on the web pages are passed to the R script. The application writes this information, as R assignments, into a text file which is concatenated with the main R script prior to execution. The application waits for the script to finish and then displays the results on the web page. A uniquely named working directory is created each time the R script is run. To pass results back to the web page, the R script writes results to this directory. The web application, on completion of the R script, displays the files in this directory on the Results page.

Running the R script

For web applications running on a Unix system the R script is run using R batch mode, the batch command being placed in a shell script:-

```
#!/bin/sh
R CMD BATCH --slave --no-restore --no-save $1 $2
```

In the Java code the shell script is run as a Process using the application's instance of the Runtime class:-

```
Runtime r = Runtime.getRuntime();
String cmd = "shell_script Rscript.R Rscript.Rout";
Process p = r.exec(cmd);
p.waitFor();
```

And for Windows systems the command-line interpreter `cmd.exe` is used to run `Rterm.exe` which runs the R script; the code being:-

```
Runtime r = Runtime.getRuntime();
String[] cmd = new String[3];
cmd[0] = "cmd.exe";
cmd[1] = "/C";
cmd[2] = "Rterm.exe --slave --no-restore --no-save Rscript.R Rscript.Rout
Process p = r.exec(cmd);
p.waitFor();
```

The Runtime class allows a Java application to interface with the environment in which it is running. A Java application has one instance of this class. The Process class has a method `waitFor()` that causes the current thread to wait until the Process has completed.

JavaScript is used to disable the 'Analyse' button while the R script is running, in order to prevent multiple submissions. However applications created by Rwui also use a synchronizer token based method [2] to ensure multiple submissions are not possible, even when JavaScript is turned off.

Passing values to the R script

Before the R script is run the values of the variables that the user entered on the web pages are passed to the R script. To do this the application writes the variables and their values, as R assignments, into a text file which is concatenated with the main R script prior to execution:

Numeric entry box If, for example, an R script variable named `my_num` was associated with a Numeric entry box when the application was created with Rwui, and the user of the completed application enters the number `1234` into this Numeric entry box, then the line `my_num <- 1234` will be added automatically to the beginning of the R script prior to execution.

Text entry box Similarly for a Text entry box; if, for example, an R script variable named `my_text` was associated with a Text entry box and the user of the completed application enters `hello` in this box, then the application will automatically add the line `my_text <- "hello"` to the beginning of the

R script. Drop-down lists, Radio buttons and Checkboxes work in a similar fashion except the choices available to the user are pre-defined.

File upload box These are dealt with in a similar fashion. If, for example, an R variable named `my_data_file` was associated with a File upload box when the application was created, then the completed web application will assign the name of the file that the user uploads with this File upload box, to the R variable `my_data_file`. For example, if the user uploads a file named `data.txt`, then the application will automatically add the line `my_data_file <- "data.txt"` to the beginning of the R script prior to execution. So in web applications created by Rwui the R script must include code to read the data file. In this example the R script needs to include a line to read the file whose name is stored in the R variable `my_data_file`, for example, `my_data <- scan(file=my_data_file)`. When the file `data.txt` is uploaded from the web page it will be stored in the correct working directory on the server, so no path to the file is required.

Displaying the results

Each time the 'Analyse' button is pressed a uniquely named working directory is created on the server. In order to pass the results generated by the R script back to the web page, the R script must write the results to files in this directory. On completion of the R script the web application looks at what files have been produced and lists them on the Results page as links, which the user can click on to view and/or download. The application also uploads any data files for processing into this directory which means that these files will also be listed as links on the Results page, giving the user the opportunity to check that the correct data has been submitted and has been uploaded correctly. And the contents of some or all of the results files can be dislayed on the Results page straight away, to save the user the trouble of having to click on a link. Which files are displayed is specified when the application is being designed in Rwui.

SessionListener

Every time a new analysis is performed, any data files will be copied to the new working directory, even if the data files have not changed between analyses. If the user presses the 'Clear Page' button then their current working directory and all the working directories from their previous submissions are deleted from the server. But if the user exits the application by closing the browser window without pressing the 'Clear Page' button, then these directories and their contents will remain on the server. To prevent the server from becoming clogged with data, the completed web application includes a SessionListener. The SessionListener detects when a session is about to expire. This usually occurs 30 minutes from when the session was last accessed, but the session timeout can be changed in the Tomcat server configuration if required (in the 'Default Session Configuration' section of the file `TOMCAT_HOME/conf/web.xml`). On detecting a session expiring the SessionListener removes all the working directories created during the session from the server. The SessionListener can be turned off by commenting out the relevant section in the file `APPLICATION_HOME/WEB-INF/web.xml`.

Validation and error messages

A feature of the Struts framework is that the web application validates data as it is entered by the user and if necessary, returns error messages to the web page. Errors that occur whilst the R program is running are also displayed. The R options command is automatically set at the beginning of the script so that if an error occurs the error message is written to a text file. The web application checks this file when the Process terminates and if it is not empty, displays the message on the web page.

References

[1] **Apache Struts** [http://struts.apache.org/].

[2] **SynchroAction** [http://www.javaworld.com/javatips/jw-javatip136.html].
