# Supplementary material for: Displaying R spatial statistics on Google dynamic maps with web applications created by Rwui
Source: Int J Health Geogr. 2012 Sep 24;11:41. doi: 10.1186/1476-072X-11-41 (PMC3548681; doi:10.1186/1476-072X-11-41)
Supplement: Additional file 3 — Documentation for R functions for writing Javascript for displaying Google dynamic maps with data overlays. [file 1476-072X-11-41-S3.pdf]

Additional File 3: Documentation for R functions for writing Javascript for displaying Google dynamic maps with data overlays

Richard Newton, Andrew Deonarine and Lorenz Wernisch

August 14, 2012

The functions can be found in the file `dynmap_functions.R` at

http://sysbio.mrc-bsu.cam.ac.uk/Rwui/tutorial/dynamic_map_tutorial/dynmap_functions.R

make.js()

An R function to construct the javascript file required by a webapp created by Rwui to display a Google dynamic map. Uses the functions `make.overlayAdd()`, `make.circlesAdd()`, `make.markersAdd()` which are also included in the file `dynmap_functions.R`.

Usage

```
make.js(filename="dynmap_sub.js",
        mapops=list(map.lat, map.lng, zoom, type="TERRAIN"),
        circlesops=list(circle.data=NULL),
        markersops=list(marker.data=NULL, iconimage=NULL),
        overlayops=list(pngname=NULL, sw=c(sw.lat, sw.lng), ne=c(ne.lat, ne.lng), def.op=50),
        kmlops = list(kmlname=NULL),
        clickable=FALSE)
```

Arguments

`filename` (String, required): Name of the javascript file associated with the dynamic map. You entered the name of this file when you created the webapp, on the page of Rwui titled ‘Enter the name of a results file to be displayed’.

`mapops` (List, required): Map options, a list containing: `map.lat` the default latitude of the map centre, `map.lng` the default longitude of the map centre, `zoom` the default zoom level and `type` the default type of map, which can be one of ‘ROADMAP’, ‘TERRAIN’, ‘SATELLITE’ or ‘HYBRID’.

`circlesops` (List, optional): For adding circle markers to the map. A list containing one item, `circle.data`, `circle.data` being a dataframe with one row for each circle to be displayed. The dataframe requires

four columns, the columns being, in order - 'latitude', 'longitude', 'colour' (expressed as the colour's CSS3 hexadecimal code `http://xilize.sourceforge.net/Reference/colorref.html`) and 'radius'.

`markersops` (List, optional): For adding Google markers to the map. A list containing two items, `markers.data` and `iconimage`. `markers.data` being a dataframe with one row for each marker to be displayed. The dataframe requires two columns, the columns being, in order - 'latitude' and 'longitude'. `iconimage` is the name of an optional png file for the icon. If `iconimage` is NULL the default Google marker icon will be used. If you require a different marker then the marker's png file must be manually copied to the top level directory of your webapp once the webapp `.war` file has been automatically unpacked by Tomcat (i.e. if your webapp is called `my_webapp` it must be copied to the directory `/TOMCAT_HOME/webapps/my_webapp/`).

`overlayops` (List, optional): For adding overlay pngs to your Google dynamic map. A list containing, `pngname` the name of the overlay's png file created by your R script, `sw` the latitude and longitude of the overlays south-west corner (a numeric vector 2 items long), `ne` the latitude and longitude of the overlays north-east corner (a numeric vector two items long), `def.op` the default opacity of the overlay (expressed as a percent). To add overlays you will need a file called `ProjectedOverlay.js` written by Sterling Udell (`http://code.google.com/p/geoxml3/`) and John Coryat (`http://www.usnaviguide.com`). This file can be downloaded from `http://code.google.com/p/geoxml3/source/browse/trunk/` and its license can be viewed here `http://www.gnu.org/copyleft/gpl.html`. Once downloaded it needs to be copied to the top level directory of your application i.e. if your webapp is called `my_webapp` then the file `ProjectedOverlay.js` needs to be copied to the directory `/TOMCAT_HOME/webapps/my_webapp/` (were you will find, for example, files such as `Results.jsp` and `EnterData.jsp`). Without the file `ProjectedOverlay.js` the overlay facility will not work.

`kmlops` (List, optional): For adding a KML file to your Google dynamic map. A list containing one item, `kmlname` the name of the KML file created by your R script. To add a KML file you will need a file called `geoxml3.js` written by Sterling Udell (`http://code.google.com/p/geoxml3/`). This file can be downloaded from `http://code.google.com/p/geoxml3/source/browse/trunk/` and its license can be viewed here `http://www.gnu.org/copyleft/gpl.html`. Once downloaded it needs to be copied to the top level directory of your application i.e. if your webapp is called `my_webapp` then the file `geoxml3.js` needs to be copied to the directory `/TOMCAT_HOME/webapps/my_webapp/` (were you will find, for example, files such as `Results.jsp` and `EnterData.jsp`). Without the file `geoxml3.js` adding the KML file will not work.

`clickable` (Boolean, optional): If a clickable dynamic map was chosen when the application was designed with Rwui (on the page 'Enter the name of a results file to be displayed') then `clickable` must be set to TRUE in order to generate the extra javascript code required for a clickable map. If a clickable map wasn't selected at the design stage `clickable` must be set to FALSE (the default).

Example

Here is a simple example of the use of the R function `make.js()`:-

Assumes `ProjectedOverlay.js` has been downloaded and copied to `/TOMCAT_HOME/webapps/YOUR_APP`. Assumes `dynmaps_functions.R` was loaded as a subsidiary file when creating the webapp and was associated at this time with an R variable called `dynmap_functions`.

The R script:-

```
source(dynmap_functions)

# Make marker data
marker.data <- data.frame(lat=c(50.965, 50.975, 50.97), lng=c(5.722, 5.75, 5.74))

# Make circle data
circle.data <- data.frame(lat=c(50.98, 50.96, 50.98), lng=c(5.73, 5.745, 5.76),
+ col=c("#0000ff","#7cfc00","#dc143c"), rad=c(10,15,20), stringsAsFactors=FALSE)

# Make a very trivial overlay

png("overlay.png", bg="transparent", type="cairo")
par(mai=c(0,0,0,0))
par(xpd=NA)
image(0,0,as.matrix(0), xaxt="n", yaxt="n", xlab=NA, ylab=NA, bty="n")
dev.off()

# Write the javascript file using make.js()

make.js(filename="dynmap_sub.js",
        mapops=list(50.975, 5.74, 14, "TERRAIN"),
        overlayops=list(pngname="overlay.png", sw=c(50.955783, 5.721112),
        + ne=c(50.992717, 5.76517), def.op=45),
        markersops=list(marker.data, iconimage=NULL),
        circlesops=list(circle.data),
        kmlops=NULL,
        clickable=FALSE)
```

A webapp using this simple example script can be found at:

http://sysbio.mrc-bsu.cam.ac.uk/dynmap_simple_example

A more realistic example of the use of `make.js()` can be seen in the R script:

http://sysbio.mrc-bsu.cam.ac.uk/Rwui/tutorial/dynamic_map_tutorial/dynmap_example.R

and a webapp using this R script can be found at:

http://sysbio.mrc-bsu.cam.ac.uk/dynmap_example
