# Supplementary material for: Displaying R spatial statistics on Google dynamic maps with web applications created by Rwui
Source: Int J Health Geogr. 2012 Sep 24;11:41. doi: 10.1186/1476-072X-11-41 (PMC3548681; doi:10.1186/1476-072X-11-41)
Supplement: Additional file 4 — Documentation on using Rwui to create a web application displaying results on a Google dynamic map. [file 1476-072X-11-41-S4.pdf]

Additional File 4: Documentation on using Rwui to create a web application displaying results on a Google dynamic map

Richard Newton, Andrew Deonarine and Lorenz Wernisch

August 14, 2012

Basics

Unlike Google Static Maps, Google Dynamic Maps can be zoomed and panned and can be switched from graphical maps to satellite images.

If you want to include a Google Dynamic Map in the webapp you are creating, on the Rwui page entitled 'Enter the name of a results file to be displayed', check the box asking whether the file to display is a dynamic geographic map (or the box asking whether the file to display is a clickable dynamic geographic map).

To actually display the dynamic map your R script will need to write a file for creating the map. This file will be written in Google Maps javascript code (see below for further details and help). You enter the name of this javascript file on the Rwui page 'Enter the name of a results file to be displayed', as the name of the results file to be displayed. Keep the name simple eg `dynmap_sub.js`. Currently you can have only one dynamic map per webapp.

Here is a basic example of the type of javascript file (`dynmap_sub.js`) the R script must write:

```
function initialize() {
    var latlng = new google.maps.LatLng(-34.397, 150.644);
    var myOptions = {
      zoom: 8,
      center: latlng,
      mapTypeId: google.maps.MapTypeId.ROADMAP
    };
    var map = new google.maps.Map(document.getElementById("map_canvas"), myOptions);
}
```

The term `map_canvas` in the above javascript code must not be changed to anything different, but apart from that any changes/additions can be made that conform to the Google Maps API:

https://developers.google.com/maps/documentation/javascript/reference.

In general the javascript file written by your R script will be different each time a user presses the analyse button and runs the R script, for example markers' positions may change etc. However there may be some javascript functions that your dynamic map requires that never change. You have the option of

uploading a file containing these javascript functions on the Rwui page ‘Upload subsidiary files’. The file must be called `dynmap_base.js`, but its variable name is immaterial provided it conforms to the naming conventions, eg. `dynmap_base` would be fine.

R functions for writing the javascript

In order to help you construct the javascript file that your R script must write in order to display your data on a Google dynamic map, R helper functions can be found in the file `dynmap_functions.R`. The file is located at:

http://sysbio.mrc-bsu.cam.ac.uk/Rwui/tutorial/dynamic_map_tutorial/dynmap_functions.R.

A convenient way of using these functions would be to download the file `dynmap_functions.R` and upload it when creating your webapp on the Rwui page ‘Upload subsidiary files’, associating the file with an R variable called, for example, `dynmap_functions`. Then include the line `source(dynmap_functions)` at the beginning of your R script and these functions will be available to subsequent commands in your R script.

The use of these functions is documented at:

http://sysbio.mrc-bsu.cam.ac.uk/Rwui/tutorial/dynamic_map_tutorial/dynmap_functions_docs/index.html.

The functions construct all the necessary javascript automatically, allowing you to display your data as circles (of different colours and radii) or as markers (with either the default Google maps icon or your own custom icon). You can of course use other features of the Google Maps API, for example polylines or polygons, but at present you will need to construct your own R functions to write the necessary javascript code, using `dynmap_functions.R` as a guideline. Alternatively the R package `plotKML` (http://cran.r-project.org/web/packages/plotKML/index.html) will write a number of R spatial classes as KML (Keyhole Markup Language). Webapps created by Rwui can display KML files on Google dynamic maps as explained in the next section.

KML file

To add a KML file to a Google dynamic map first your R script needs to create and write the KML to a file. The R package `plotKML` (http://cran.r-project.org/web/packages/plotKML/index.html) which will write a number of R spatial classes as a KML file is particularly useful for doing this.

Your R script then needs to specify that the KML file it has written must be displayed on the map, which can be done using the R function `make.js()` which can be found in the file

http://sysbio.mrc-bsu.cam.ac.uk/Rwui/tutorial/dynamic_map_tutorial/dynmap_functions.R.

Here is a usage example:

```
make.js(filename="dynmap_sub.js",
        mapops=list(50.975, 5.74, 14, "TERRAIN"),
```

```
kmlops = list(kmlname="my_kml_file.kml"),
overlayops=list(pngname="overlay.png", sw=c(50.96, 5.73), ne=c(50.99, 5.76), def.op=45),
markersops=list(marker.data, iconimage=NULL),
circlesops=list(circle.data),
clickable=FALSE)
```

You will also require a file called geoxml3.js written by Sterling Udell (http://code.google.com/p/geoxml3/). This file can be downloaded from http://code.google.com/p/geoxml3/source/browse/trunk/ and its license can be viewed here http://www.gnu.org/copyleft/gpl.html. Once downloaded it needs to be copied to the top level directory of your application i.e. if your webapp is called my_webapp then geoxml3.js needs to be copied to the directory /TOMCAT_HOME/webapps/my_webapp/ (were you will find, for example, files such as Results.jsp and EnterData.jsp). Without the file geoxml3.js adding a KML file will not work.

Please note: The first line of a KML file produced by the current version of plotKML (Version 0.2-2) reads:

```
"<kml xmlns:xsd=\"http://schemas.opengis.net/kml/2.2.0/ogckml22.xsd\"
     xmlns:xmlns=\"http://www.opengis.net/kml/2.2/\" version=\"1.0\">"
```

One section in the above line, `xmlns:xmlns=\"http://www.opengis.net/kml/2.2/\"`, stops the KML file being displayed with Google dynamic maps on Rwui webapps. Therefore your R code, once it has produced the KML file using plotKML needs to remove this text from the first line of the KML file. Here is an example of how to do this:

```
tmp <- readLines("my_kml_file.kml")
tmp[1]<-sub("xmlns:xmlns=\"http://www.opengis.net/kml/2.2/\"", "", tmp[1])
writeLines(tmp, con="my_kml_file.kml")
```

png Overlays

You can also display an overlay on the map, that is a png image which is displayed on top of the Google map with a variable degree of transparency so that details of the underlying map can still be seen. To use the overlay facility you will require a file called ProjectedOverlay.js written by Sterling Udell (http://code.google.com/p/geoxml3/) and John Coryat (http://www.usnaviguide.com>). This file can be downloaded from http://code.google.com/p/geoxml3/source/browse/trunk/ and its license can be viewed here http://www.gnu.org/copyleft/gpl.html. Once downloaded it needs to be copied to the top level directory of your application i.e. if your webapp is called my_webapp then ProjectedOverlay.js needs to be copied to the directory /TOMCAT_HOME/webapps/my_webapp/ (were you will find, for example, files such as Results.jsp and EnterData.jsp). Without the file ProjectedOverlay.js the overlay facility will not work. (Windows users: care should be taken that downloading doesn't surreptitiously add an extra extension to the filename eg. ProjectedOverlay.js.txt will not work. If in doubt, copy and paste the code from the web page into a file called ProjectedOverlay.js).

creating png files

The R command `png()` allows you to create png files with transparent backgrounds which is just what you need for an overlay. `png` uses 'cairo', 'Xlib' or 'quartz' to create the `png` so R does need to be compiled with support for at least one of these in order to create the overlay successfully. And for `type = "Xlib"` `png()` may not be usable unless the X11 display is available to the owner of the R process. `type = "cairo"` requires cairo 1.2 or later.

If `png()` is not supported you could use `bitmap()` to create the overlay:

```
bitmap(file="overlay.png")
... plot the overlay  ...
dev2bitmap(file="overlay.png")
dev.off()
```

however the background will not be transparent. `bitmap` requires 'ghostscript' to be installed.

API key

All Google Maps API applications should use an API key. The simple steps for obtaining a key can be found at https://developers.google.com/maps/documentation/javascript/tutorial#api_key.

Once you have a key here is how to use it:-

- First install your application i.e. copy `YOUR_WEBAPP.war` to `/TOMCAT_HOME/webapps`.
- Wait for Tomcat to unpack the war file, then go to directory `/TOMCAT_HOME/webapps/YOUR_WEBAPP`
- Open file `EnterData.jsp` in an editor
- Find the line:

  ```
  src="http://maps.googleapis.com/maps/api/js?key=YOUR_API_KEY&amp;sensor=false&amp;libraries=geometry"
  ```

- Replace `YOUR_API_KEY` with your api key, and save and close the file.
- Do the same edit for the file `Results.jsp`

If you don't yet have an API key, and want to first test things out without one, then just remove the characters `key=YOUR_API_KEY&` from the above line in both `EnterData.jsp` and `Results.jsp`.

Examples

The documentation for the `dynamic_functions.R` at:

http://sysbio.mrc-bsu.cam.ac.uk/Rwui/tutorial/dynamic_map_tutorial/dynmap_functions_docs/index.html

includes a short R script called `dynmap_simple_example.R` as an example of the use of the `dynmap_functions.R`. The R script can be downloaded here:

http://sysbio.mrc-bsu.cam.ac.uk/Rwui/tutorial/dynamic_map_tutorial/dynmap_simple_example.R

and a webapp created by Rwui to run this script can be found here:

http://sysbio.mrc-bsu.cam.ac.uk/dynmap_simple_example.

A step-by-step guide to recreating this simple example web application is given in the file:

http://sysbio.mrc-bsu.cam.ac.uk/Rwui/tutorial/dynamic_map_tutorial/dynmap_step_by_step/index.html.

A more realistic example application can be found here:

http://sysbio.mrc-bsu.cam.ac.uk/dynmap_example

This application runs the example kriging code by Edzer Pebesma, Roger Bivand and others from the 'sp' package (http://cran.r-project.org/web/packages/sp/index.html) and 'gstat' package (http://www.gstat.org) on the Meuse river data set (M G J Rikken and R P G Van Rijn, 1993. Soil pollution with heavy metals in the floodplains of the Meuse. Doctoraalveldwerkverslag, Utrecht University)

Here are links to a csv data file that can be downloaded and analysed with this application:

http://sysbio.mrc-bsu.cam.ac.uk/Rwui/tutorial/dynamic_map_tutorial/meuse_zinc.csv

The complete application tgz as created by Rwui can be found here:

http://sysbio.mrc-bsu.cam.ac.uk/Rwui/tutorial/dynamic_map_tutorial/dynmap_example.tgz

And here is a link to the R code the web application is running:

http://sysbio.mrc-bsu.cam.ac.uk/Rwui/tutorial/dynamic_map_tutorial/dynmap_example.R

A step-by-step guide to recreating this example web application is given in the second section of the file:

http://sysbio.mrc-bsu.cam.ac.uk/Rwui/tutorial/dynamic_map_tutorial/dynmap_step_by_step/index.html.

Projection system

Please note that depending on how an overlay was produced it may need to be converted to the projection used by Google Maps. Simply setting the SW and NE lat/longs of the overlay is not sufficient because, if the overlay was generated in a different projection system, it will be warped in the Google Maps projection.

Fortunately the R package 'raster' (http://cran.r-project.org/web/packages/raster/) has simple to use functions for changing a raster of values from one projection system to another. 'raster' uses the R package 'rgdal' http://cran.r-project.org/web/packages/rgdal/ to do this, and 'rgdal' itself requires the PROJ.4 library (http://trac.osgeo.org/proj) and GDAL (http://www.gdal.org/) to be installed.

Here is a simple example, XYZ values in the widely used WGS84 projection system (EPSG code: 4326) are converted to a raster and projected into the Google Maps projection system (EPSG code: 3857):

```
r.utm <- rasterFromXYZ(my.xyz.data)
projection(r.utm) <- "+init=epsg:4326"
```

```
r.goog <- projectRaster(r.utm,  crs="+init=epsg:3857")
```

Further examples of the use of the ‘raster’ package are contained in the sample code:

http://sysbio.mrc-bsu.cam.ac.uk/Rwui/tutorial/dynamic_map_tutorial/dynmap_example.R

and

http://sysbio.mrc-bsu.cam.ac.uk/Rwui/tutorial/dynamic_map_tutorial/click_dynmap_example.R.

There is an interesting article about the Google Maps projection system ‘The Google Maps / Bing Maps Spherical Mercator Projection’ at:

http://alastaira.wordpress.com/2011/01/23/the-google-maps-bing-maps-spherical-mercator-projection

Creating a ‘clickable’ dynamic geographic map

When creating the webapp, on the Rwui page entitled ‘Enter the name of a results file to be displayed’, you can specify the results are to be displayed as a ‘clickable’ dynamic geographic map. If selected, the geographic map will be ‘clickable’, in that a left click will generate a region of interest (ROI). The region of interest can be either a circle, irregular polygon, square or rectangle. The shapes are all draggable and resizable and removable. When the ‘Analyse’ button is pressed the coordinates of the ROI are automatically made available to your R script. In this way users of the webapp can define regions of interest on the geographic map for analysis by the R script. Radio buttons below the map select which shape a left click will generate.

If the ‘Circle’ radio button is selected then a left-click on the map generates a circle which can be dragged using the central marker, resized using the edge marker and removed by a right-click on the central marker. If the ‘Polygon’ radio button is selected then a left-click on the map generates a polygon vertex. You can add as many vertices as you like but you need at least three. Each vertex can be dragged by the vertex marker and a single click on a marker will remove it. The entire polygon can be dragged by any edge and removed by a double click. If the ‘Square’ radio button is selected then a left-click on the map generates a square which can be dragged by any edge and resized using the corner marker and removed by a right click on the square. If the ‘Rectangle’ radio button is selected then a left-click on the map generates a rectangle which can be dragged by any edge and resized using any marker and removed by a right click on the rectangle. Currently users can just generate one circle, polygon, square or rectangle.

The coordinates and area of the shape are displayed below the map. Then when the ‘Analyse’ button is pressed the coordinates of the region of interest are automatically made available to your R script. This is accomplished by the following lines being automatically added to the beginning of your R script prior to it running:

```
polylats <- c()   # contains the latitudes of the polygon vertices (if any)
polylngs <- c()   # contains the longitudes of the polygon vertices (if any)
circpos <- c()    # contains the latitude and longitude of the centre of the circle (if any)
circrad <- c()    # contains the radius of the centre of the circle in kilometres (if any)
sqlats <- c()     # contains the lats of the SW and NE corners of the square (if any)
sqlngs <- c()     # contains the longs of the SW and NE corners of the square (if any)
```

```
rectlats <- c()   # contains the lats of the SW and NE corners of the rectangle (if any)
rectlngs <- c()   # contains the longs of the SW and NE corners of the rectangle (if any)
roishape <- ""    # contains the type of shape: "circ", "square", "rect", "poly" or ""
mappars <- c()    # contains the map's centre lat, centre lng and zoom level
```

Since the above input variable names are already in use by the webapp to transfer information from the web page to the R script, it is of course important that none of theses ten names are used as input variables of your R script (those R script variables that are assigned by the user on the web page).

In order to create a 'clickable' dynamic geographic map, besides specifying this when creating the webapp with Rwui, your R script needs to generate the correct javascript code. This can simply be done using the R function `make.js(..., clickable=TRUE)`, which is provided in the file:

http://sysbio.mrc-bsu.cam.ac.uk/Rwui/tutorial/dynamic_map_tutorial/dynmap_functions.R

and documented at:

http://sysbio.mrc-bsu.cam.ac.uk/Rwui/tutorial/dynamic_map_tutorial/dynmap_functions_docs/index.html.

Download the file `dynmap_functions.R` and upload it when creating your webapp on the Rwui page 'Upload subsidiary files', associating the file with an R variable called, for example, `dynmap_functions`. Then include the line `source(dynmap_functions)` at the beginning of your R script and the function `make.js()` will be available in your R script.

The region of interest from a submission will also be displayed on the geographic map on the submission's 'Results' page, but on the 'Results' page the shape will not be draggable, resizable or removable. The region of interest is ony draggable, resizable or removable on the geographic map on the main web page of the application.

An example application can be found here:

http://sysbio.mrc-bsu.cam.ac.uk/click_dynmap_example

The R code the web application is running:

http://sysbio.mrc-bsu.cam.ac.uk/Rwui/tutorial/dynamic_map_tutorial/click_dynmap_example.R

Acknowledgements

The 'clickable' dynamic map uses javascript code written by Wolfgang Pichler:

http://www.wolfpil.de/v3/flexible-polygon.html (polygons)

and Luke Mahe: https://developers.google.com/maps/articles/mvcfun (circles).
