# Supplementary material for: Displaying R spatial statistics on Google dynamic maps with web applications created by Rwui
Source: Int J Health Geogr. 2012 Sep 24;11:41. doi: 10.1186/1476-072X-11-41 (PMC3548681; doi:10.1186/1476-072X-11-41)

Additional File 5: Step-by-step guide to using Rwui to create webapps that display R script results on Google dynamic maps

Richard Newton, Andrew Deonarine and Lorenz Wernisch

July 7, 2012

1 Simple example

We are going to create and install a web application to run an R script `dynmap_simple_example.R`. The web application will display a Google dynamic map with a simple overlay created by the R script. The completed web application can be seen at:

http://sysbio.mrc-bsu.cam.ac.uk/dynmap_simple_example.

1.1 The R script

First download the R script:

http://sysbio.mrc-bsu.cam.ac.uk/Rwui/tutorial/dynamic_map_tutorial/dynmap_simple_example.R

Or copy and paste the following to a file:

```
source(dynmap_functions)

# Make marker data
marker.data <- data.frame(lat=c(50.965, 50.975, 50.97), lng=c(5.722, 5.75, 5.74))

# Make circle data
circle.data <- data.frame(c(50.975, 50.96, 50.98), c(5.725, 5.73, 5.76), c("#0000FF","#FFD700","#DC143C"),
                          c(50,150,90), stringsAsFactors=FALSE)

# Make toy overlay

png("overlay.png",  bg="transparent", type="cairo")
par(mai=c(0,0,0,0))
par(xpd=NA)
image(0,0,as.matrix(0), xaxt="n", yaxt="n", xlab=NA, ylab=NA, bty="n")
dev.off()

make.js(fname="dynmap_sub.js",
        mapops=list(50.975, 5.74, 14, "TERRAIN"),
        overlayops=list(pngname="overlay.png", sw=c(50.965783, 5.731112),
                        ne=c(50.992717, 5.76517), def.op=45),
        markersops=list(marker.data, iconimage=NULL),
```

```
circlesops=list(circle.data),
clickable=FALSE)
```

1.2 Helper functions

You will also need to download the file `dynmap_functions.R` from:

http://sysbio.mrc-bsu.cam.ac.uk/Rwui/tutorial/dynamic_map_tutorial/dynmap_functions.R

1.3 Creating the webapp with Rwui

- Go to Rwui at: http://sysbio.mrc-bsu.cam.ac.uk/Rwui and click `Start Rwui`
- **Enter a title for the application**

  Type `Dynamic map simple example` in the top box and click `Enter`
- **Enter an introductory explanation (optional)**

  Leave blank and click `Enter`
- **Include further instructions (optional)**

  Leave blank and click `Enter`
- **Choose a variable input item**

  Ignore and click `Finished composing page`
- **Validation**

  Leave as default and click `Enter`
- **Enter a name for the application**

  Type `dynmap_simple_example` and click `Enter`
- **Enter the name of a results file to be displayed (optional)**

  Type `dynmap_sub.js`

  Tick the checkbox by 'Tick box if file is a dynamic geographic map'

  Click `Enter`

  Click `Finished entering filenames`
- **Enter the name of a list of results files (optional)**

  Ignore and click `Finished entering filenames`
- **Layout of results files**

  Leave as default and click `Enter`

- **Number of columns in the layout tables**

  Leave as default and click `Enter`

- **Display results on the analysis page?**

  Tick check box by 'Tick box to display results on the analysis page' and click `Enter`

- **Process information**

  Ignore and click `Enter`

- **Graphical process information**

  Ignore and click `Enter`

- **Upload R script**

  Browse to location where you have saved the downloaded R script `dynmap_simple_example.R` and click `Enter`

- **Upload subsidiary R scripts and data sources (optional)**

  In the top box type `dynmap_functions`

  Browse to location where you have saved the downloaded file `dynmap_functions.R` and click `Enter`

  Click `Finished entering subsidiary files`

- **Add an initial Login page?**

  Leave as default and click `Enter`

- **Which way to return results to user?**

  Leave as default and click `Enter`

- **Include a Cancel button on the web page**

  Ignore and click `Enter`

- **Create application**

  Click `Create application`

- **Completed**

  Click `dynmap_simple_example.tgz` (or `dynmap_simple_example.zip`, unpacked they are identical) and save file to your PC

- You are now finished with Rwui.

1.4 Installing Java

- Download the Java SE Runtime Environment (JRE) from:

  http://www.oracle.com/technetwork/java/javase/downloads/index.html

- Install the JRE on your PC according to the instructions included with the release, or linked to on the above page.
- Set an environment variable named `JRE_HOME` to the pathname of the directory into which you installed the JRE:

  On Windows if the path is e.g. `c:\jre6.0`, then

  – Right click on the My Computer icon on your desktop and select properties
  – Click the Advanced Tab
  – Click the Environment Variables button
  – Under System Variable, click New
  – Enter the variable **name** as JRE_HOME
  – Enter the variable **value** as the install path for the JRE e.g. `c:\jre6.0`
  – Click OK
  – Click Apply Changes

  Or on Linux if the path is e.g. `/usr/local/java/jre6.0` then

  – Add the line `export JRE_HOME=/usr/local/java/jre6.0` to file `.bash_profile`
  – Save
  – Run `source ~/.bash_profile`
- You may also use the full JDK rather than just the JRE. In this case set the `JAVA_HOME` environment variable to the pathname of the directory into which you installed the JDK, e.g. `c:\jdk6.0` or `/usr/local/java/jdk6.0`.

1.5 Installing Tomcat

- Download a binary distribution of Tomcat from:

  http://tomcat.apache.org
- Unpack the binary distribution into a convenient location on your PC.
- This will create a directory called eg. `apache-tomcat-6.0.35`.
- NB. On Windows - install into a directory called, for example, `C:\tomcat\apache-tomcat-6-0-35`. The path must not have any spaces, which prevent applications from running. Dots in the directory names also cause problems, as might other characters, so just use alphanumerics and hyphens as shown in the example.
- From here on we will refer to the path to Tomcat (eg `/usr/local/apache-tomcat-6.0.35` or `c:\tomcat\apache-tomcat-6-0-35`) as `TOMCAT_HOME`

- Tomcat can be started by executing the following commands:

  `\TOMCAT_HOME\bin\startup.bat` (Windows)

  `/TOMCAT_HOME/bin/startup.sh` (Linux)

- NB. On Linux make sure `startup.sh` is executable first
- Open a browser and type http://localhost:8080 in the address bar and this should take you to Tomcat's home page on your machine, indicating Tomcat is installed correctly and working.

1.6 Path to R

1.6.1 Linux

Nothing needs to be done on a Linux machine.

1.6.2 Windows

Need to set the path to R's bin directory

- Right click on the My Computer icon on your desktop and select properties
- Click the Advanced Tab
- Click the Environment Variables button
- In the 'System Variables' box scroll down and select variable 'Path' and press 'Edit'
- Add the path to R's bin directory by adding eg. `C:\Program Files\R\R-2.12.0\bin` to the ';' separated list
- Click OK
- Click Apply Changes

1.7 Installing the web application

- If you downloaded the tgz file unpack it: `tar zxvf dynmap_simple_example.tgz`
- Or if you downloaded the zip file unpack it: `unzip dynmap_simple_example.zip`
- A directory called `dynmap_simple_example` will have been created by the unpacking
- Copy the file `dynmap_simple_example/deploy/dynmap_simple_example.war` to the directory:

  `/TOMCAT_HOME/webapps`

- Tomcat should unpack the `.war` file automatically, but if not simply stop and start Tomcat and the `.war` file should unpack.

  (Tomcat can be stopped by executing the following command:

\TOMCAT_HOME\bin\shutdown (Windows)

/TOMCAT_HOME/bin/shutdown.sh (Unix))

- This will give a directory /TOMCAT_HOME/webapps/dynmap_simple_example

1.8 ProjectedOverlay.js

- Download file ProjectedOverlay.js from:

  http://code.google.com/p/geoxml3/source/browse/trunk/

- Copy file ProjectedOverlay.js to directory /TOMCAT_HOME/webapps/dynmap_simple_example/
- Windows users note: care should be taken that downloading doesn't surreptitiously add an extra extension to the filename eg. ProjectedOverlay.js.txt will not work. If in doubt copy and paste the code from the web page into a file called ProjectedOverlay.js.

1.9 Sorting out the API key

- Follow the instructions for obtaining an Google Maps API key at:

  https://developers.google.com/maps/documentation/javascript/tutorial#api_key

- Then, in a text editor, open file /TOMCAT_HOME/webapps/dynmap_simple_example/EnterData.jsp
- Locate the line (approx. line 31) reading:

  src="http://maps.googleapis.com/maps/api/js?key=YOUR_API_KEY&sensor=false&libraries=geometry"

- Replace YOUR_API_KEY with the api key you have just obtained from Google.
- (Note: if you don't currently have an API key you can simply remove the characters key=YOUR_API_KEY& from the above line)
- Do the same for file /TOMCAT_HOME/webapps/dynmap_simple_example/Results.jsp

1.10 Using the web application

- Open a browser and type http://localhost:8080/dynmap_simple_example in the address bar.
- You should see your webapp, the same as http://sysbio.mrc-bsu.cam.ac.uk/dynmap_simple_example
- Click Analyse
- You should see the Google dynamic map with overlay as shown in Figure 1.
- The opacity of the png overlay can be varied by the controls below the map.
- The map can be zoomed and panned and the type changed to satellite etc using the usual Google map controls.

1.11 Technical note

Note that the R script uses `png()` to create the overlay. `png()` allows you to create png files with transparent backgrounds which is just what you need for an overlay. `png` uses 'cairo', 'Xlib' or 'quartz' to create the png so R does need to be compiled with support for at least one of these in order to create the overlay successfully. And for `type = "Xlib"`, `png()` may not be usable unless the X11 display is available to the owner of the R process. `type = "cairo"` requires cairo 1.2 or later.

If `png()` is not supported, as a temporary measure you could substitute the following code to create the overlay:

```
bitmap(file="overlay.png")
par(mai=c(0,0,0,0))
par(xpd=NA)
image(0,0,as.matrix(0), xaxt="n", yaxt="n", xlab=NA, ylab=NA, bty="n")
dev2bitmap(file="overlay.png")
dev.off()
```

The background will not be transparent but since this simple overlay has no background areas, in this case it doesn't matter. `bitmap` requires 'ghostscript' to be installed.

Figure 1: Screenshot of the simple example application http://sysbio.mrc-bsu.cam.ac.uk/dynmap_simple_example

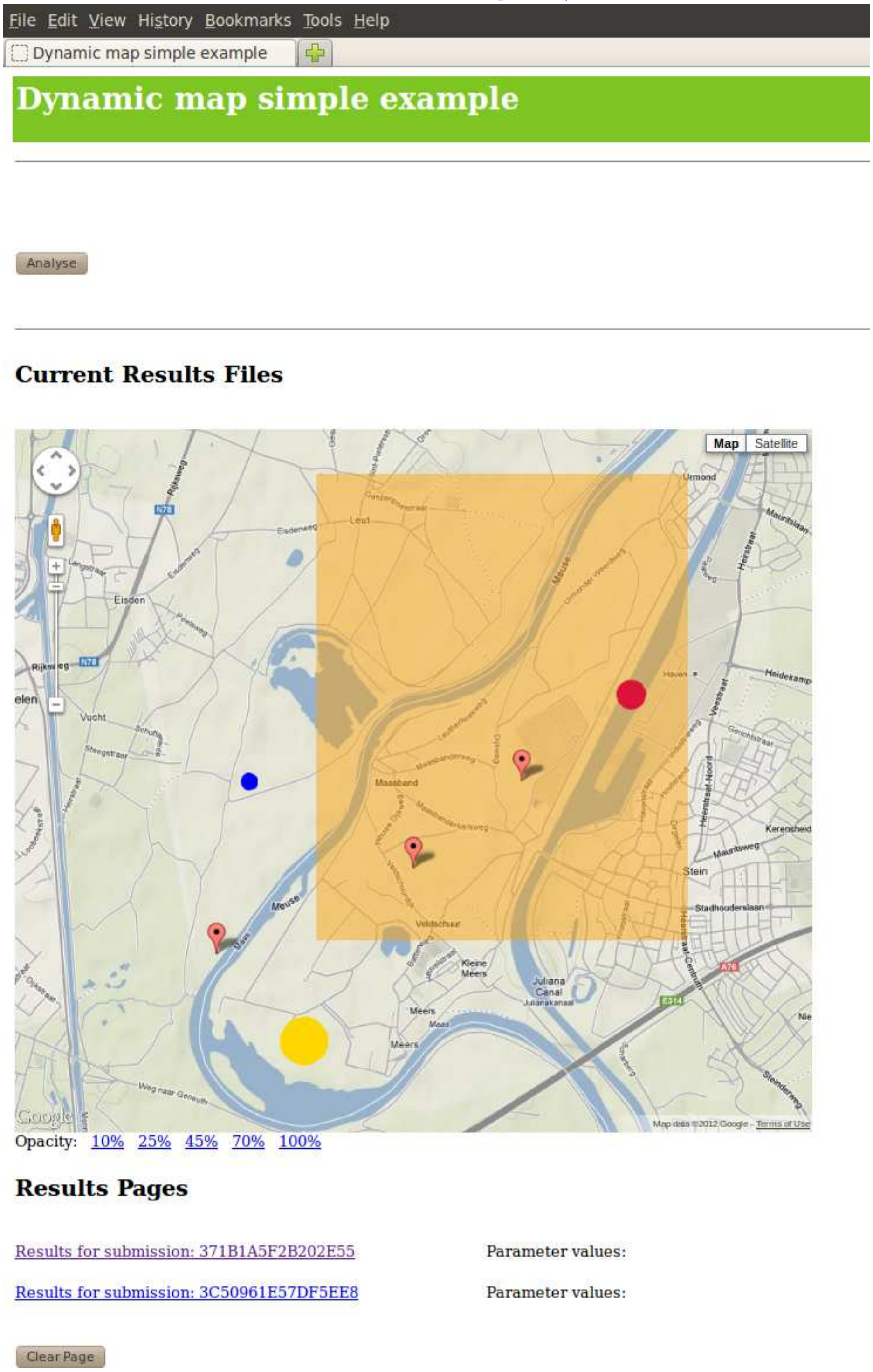

2 Meuse river example

We are going to create and install a web application to run an R script `dynmap_example.R`. This application runs the example kriging code from the R package sp (Pebesma E, Bivand R: sp: classes and methods for spatial data http://CRAN.R-project.org/web/packages/sp/), on the Meuse River soil pollution data (Rikken MGJ, Van Rijn RPG: Soil pollution with heavy metals in the floodplains of the Meuse. Doctoraalveldwerkverslag, Utrecht University 1993). The completed web application can be seen at:

http://sysbio.mrc-bsu.cam.ac.uk/dynmap_example.

2.1 The R script

First download the R script:

http://sysbio.mrc-bsu.cam.ac.uk/Rwui/tutorial/dynamic_map_tutorial/dynmap_example.R

Or copy and paste the following to a file:

```
source(dynmap_functions)
library(sp)
library(gstat)
library(rgdal)
library(raster)
load(meuse_grid)
load(meuse_grid_ll)

meuse <- read.csv(file=datafile)
coordinates(meuse)=~x+y
coordinates(meuse.grid) = ~x+y
gridded(meuse.grid) = TRUE

## make circle.data

circle.data <- data.frame()
if(markers != "none"){
    for(j in 1:nrow(meuse)){
        dt <- coordinates(meuse.grid_ll)[which.min((coordinates(meuse.grid)[,1]-coordinates(meuse)[j,1])**2 +
              (coordinates(meuse.grid)[,2]-coordinates(meuse)[j,2])**2),]
        circle.data[j,1] <- dt[2]
        circle.data[j,2] <- dt[1]
        bns <- quantile(meuse@data[,"z"], probs = seq(0, 1, 1/5))
        if(markers=="circles"){
            if(meuse@data[j,"z"]<=bns[2]){circle.data[j,3] <- "#000000"}
            if(meuse@data[j,"z"]>bns[2] && meuse@data[j,"z"]<=bns[3]){circle.data[j,3] <- "#0000FF"}
            if(meuse@data[j,"z"]>bns[3] && meuse@data[j,"z"]<=bns[4]){circle.data[j,3] <- "#9932CC"}
            if(meuse@data[j,"z"]>bns[4] && meuse@data[j,"z"]<=bns[5]){circle.data[j,3] <- "#FF8C00"}
            if(meuse@data[j,"z"]>bns[5]){circle.data[j,3] <- "#FFFF00"}
            circle.data[j,4] <- 15
        }
        if(markers=="bubbles"){
            circle.data[j,3] <- "#FF0000"
            if(meuse@data[j,"z"]<=bns[2]){circle.data[j,4]<- 5}
```

```
            if(meuse@data[j,"z"]>bns[2] && meuse@data[j,"z"]<=bns[3]){circle.data[j,4] <- 10}
            if(meuse@data[j,"z"]>bns[3] && meuse@data[j,"z"]<=bns[4]){circle.data[j,4] <- 20}
            if(meuse@data[j,"z"]>bns[4] && meuse@data[j,"z"]<=bns[5]){circle.data[j,4] <- 30}
            if(meuse@data[j,"z"]>bns[5]){circle.data[j,4] <- 40}
        }
    }
}else{
    circle.data <- NULL
}
circlesops <- list(circle.data)


### Krige and make overlay

overlayops <- NULL

if(kri != "none"){
    v.ok = variogram(log(z)~1, meuse)
    ok.model = fit.variogram(v.ok, vgm(1, "Exp", 500, 1))
    v.uk = variogram(log(z)~sqrt(dist), meuse)
    uk.model = fit.variogram(v.uk, vgm(1, "Exp", 300, 1))
    meuse[["ff"]] = factor(meuse[["ffreq"]])
    meuse.grid[["ff"]] = factor(meuse.grid[["ffreq"]])
    v.sk = variogram(log(z)~ff, meuse)
    sk.model = fit.variogram(v.sk, vgm(1, "Exp", 300, 1))
    if(kri=="ordinary"){
        kg = krige(log(z)~1, meuse, meuse.grid, model = ok.model)
    }
    if(kri=="universal"){
        kg = krige(log(z)~sqrt(dist), meuse, meuse.grid, model = uk.model)
    }
    if(kri=="stratified"){
        kg = krige(log(z)~ff, meuse, meuse.grid, model = sk.model)
    }
    kg[["se"]] = sqrt(kg[["var1.var"]])
    if(disp == "prediction"){
        dat <- cbind(kg@coords, kg@data[,1])
    }
    if(disp == "se"){
        dat <- cbind(kg@coords, kg@data[,3])
    }

    ## Project the data from Rijksdriehoek (RDH) (Netherlands topographical) map coordinates to google map coordinates

    r<-rasterFromXYZ(dat)
    projection(r)<-paste("+init=epsg:28992","+towgs84=565.237,50.0087,465.658,-0.406857,0.350733,-1.87035,4.0812")
    r.goog <- projectRaster(r,  crs="+init=epsg:3857")

    ## Find the UTM lat/longs of the corners of the google maps coordinates raster

    r.utm.ext <- projectExtent(r.goog,  crs="+init=epsg:4326")

    sw <- c(ymin(r.utm.ext), xmin(r.utm.ext))
    ne <- c(ymax(r.utm.ext), xmax(r.utm.ext))

    ## Create overlay png
```

```
    png("overlay.png", type="cairo", bg="transparent", width=5*ncol(r.goog), height=5*nrow(r.goog),res=72)
    par(mai=c(0,0,0,0))
    par(xpd=NA)
    image(rotate.image(as.matrix(r.goog)), main="", bty="n", xaxt="n", yaxt="n", col=topo.colors(30))
    dev.off()
    overlayops <- list(pngname="overlay.png", sw=sw, ne=ne, def.op=45)
}

make.js("dynmap_sub.js", mapops=list(50.975, 5.74, 14, "TERRAIN"), overlayops=overlayops, circlesops=circlesops)


### Make title and legend

if(kri=="none"){
   title <- chem
}else{
   if(disp=="prediction"){
       title <- paste(chem, " - ", kri, " kriging", " - ", "prediction", sep="")
   }else{
       title <- paste(chem, " - ", kri, " kriging", " - ", "standard errors", sep="")
   }
}
if(markers == "circles"){
    legend <- c(paste("<", bns[2], sep=" "),
                paste(bns[2], "-", bns[3], sep=" "),
                paste(bns[3], "-", bns[4], sep=" "),
                paste(bns[4], "-", bns[5], sep=" "),
                paste(">", bns[5], sep=" "))
    cols <- c("#000000","#0000FF", "#9932CC", "#FF8C00", "#FFFF00")
}
if(markers == "bubbles"){
    legend <- c(paste("<", bns[2], sep=" "),
                paste(bns[2], "-", bns[3], sep=" "),
                paste(bns[3], "-", bns[4], sep=" "),
                paste(bns[4], "-", bns[5], sep=" "),
                paste(">", bns[5], sep=" "))
    pt.cex <- c(5/6,10/6,20/6,30/6,40/6)
    cols <- c("#FF0000")
}

png("legend.png", type="cairo", width=750, height=750,res=72)
if(kri != "none"){
    rng <- range(as.matrix(r.goog), na.rm=T)
    lz <- seq(rng[1],rng[2], len=100)
    legend.z <- NULL
    for(i in 1:10){
        legend.z <- rbind(legend.z, lz)
    }
    image.default(1:10, lz, legend.z, main = title, xlim=c(1,100),  cex.main=2, cex.axis=2, xlab=NA, ylab=NA,
                  bty="n", xaxt="n", col=topo.colors(30))
}else{
    plot(0,1, main=title, xlab=NA, ylab=NA, bty="n", xaxt="n", yaxt="n", type="n", cex.main=2)
}
if(markers == "circles"){
    legend("center", legend=legend,  pch=19, col=cols, title="Measurements", cex=2, y.intersp=1.5)
```

```
}
if(markers == "bubbles"){
    legend("center", legend=legend,  pch=19, pt.cex=pt.cex, col=cols, title="Measurements",
           cex=2, y.intersp=1.5)
}
dev.off()
```

This web application also requires two `.Rdata` files, `meuse_grid.Rdata` and `meuse_grid_ll.Rdata`, which you will upload as subsidiary files when creating the application with Rwui. You can download the two files at:

http://sysbio.mrc-bsu.cam.ac.uk/Rwui/tutorial/dynamic_map_tutorial/meuse_grid.Rdata

http://sysbio.mrc-bsu.cam.ac.uk/Rwui/tutorial/dynamic_map_tutorial/meuse_grid_ll.Rdata

2.2 Helper functions

You will also need to download the file `dynmap_functions.R` from:

http://sysbio.mrc-bsu.cam.ac.uk/Rwui/tutorial/dynamic_map_tutorial/dynmap_functions.R

2.3 Creating the webapp with Rwui

- Go to Rwui at: http://sysbio.mrc-bsu.cam.ac.uk/Rwui and click `Start Rwui`
- **Enter a title for the application**

  Type `Dynamic map example` in the top box and click `Enter`
- **Enter an introductory explanation (optional)**

  Paste the following in the box:

```
This demonstration application runs the example kriging code from the
<A href=http://cran.r-project.org/web/packages/sp/index.html target="_blank">sp</A> package of
Edzer Pebesma, Roger Bivand and others, on the data of M G J Rikken and R P G Van Rijn
(Soil pollution with heavy metals in the floodplains of the Meuse, Utrecht University, 1993).

<P>

Here are some data files that can be downloaded for analysis by this webapp:

<P>

<ul>
<li>
<A href=http://sysbio.mrc-bsu.cam.ac.uk/Rwui/tutorial/dynamic_map_tutorial/meuse_zinc.csv>Zinc</A>
</li>
<li>
<A href=http://sysbio.mrc-bsu.cam.ac.uk/Rwui/tutorial/dynamic_map_tutorial/meuse_copper.csv>Copper</A>
</li>
<li>
<A href=http://sysbio.mrc-bsu.cam.ac.uk/Rwui/tutorial/dynamic_map_tutorial/meuse_cadmium.csv>Cadmium</A>
```

```
</li>
<li>
<A href=http://sysbio.mrc-bsu.cam.ac.uk/Rwui/tutorial/dynamic_map_tutorial/meuse_lead.csv>Lead</A>
</li>
</ul>
```

Click `Enter`

- **Include further instructions (optional)**

  Leave blank and click `Enter`

- **Choose a variable input item**

  - Select `Section heading` from the drop-down list and click `Enter`

    Type `Enter name of pollutant` in the top box and click `Enter`
  - Select `Text box` from the drop-down list and click `Enter`

    Type `chem` in the top box and click `Enter`
  - Select `Section heading` from the drop-down list and click `Enter`

    Type `Upload data file` in the top box and click `Enter`
  - Select `File Upload Box` from the drop-down list and click `Enter`

    Type `datafile` in the top box and click `Enter`
  - Select `Section heading` from the drop-down list and click `Enter`

    Type `Display measurement points` in the top box and click `Enter`
  - Select `Drop-down List` from the drop-down list and click `Enter`

    Type `markers` in the top box and click `Enter`

    Leave on default radio-button (Text) and click `Enter`

    Type `circles` and click `Enter`

    Type `bubbles` and click `Enter`

    Type `none` and click `Enter`

    Click `Finished entering list entries`
  - Select `Section heading` from the drop-down list and click `Enter`

    Type `Display Kriging` in the top box and click `Enter`
  - Select `Drop-down List` from the drop-down list and click `Enter`

    Type `kri` in the top box and click `Enter`

    Leave on default radio-button (Text) and click `Enter`

    Type `none` and click `Enter`

Type `ordinary` and click `Enter`

Type `universal` and click `Enter`

Type `stratified` and click `Enter`

Click `Finished entering list entries`

– Select `Section heading` from the drop-down list and click `Enter`

Type `Display prediction or standard errors` in the top box

Check the lowest radio-button to reduce the font size.

Click `Enter`

– Select `Drop-down List` from the drop-down list and click `Enter`

Type `disp` in the top box and click `Enter`

Leave on default radio-button (Text) and click `Enter`

Type `prediction` and click `Enter`

Type `se` and click `Enter`

Click `Finished entering list entries`

– Click `Finished composing page`

- **Validation**

  Leave as default and click `Enter`

- **Enter a name for the application**

  Type `dynmap_example` and click `Enter`

- **Enter the name of a results file to be displayed (optional)**

  Type `dynmap_sub.js` in the top box

  Tick the checkbox by ‘Tick box if file is a dynamic geographic map’

  Change the ‘Width’ to 750

  Click `Enter`

  Type `legend.png` in the top box

  DO NOT Tick the checkbox by ‘Tick box if file is a dynamic geographic map’

  Change the ‘Width’ to 750

  Click `Enter`

  Click `Finished entering filenames`

- **Enter the name of a list of results files (optional)**

  Ignore and click `Finished entering filenames`

- **Layout of results files**

  Leave as default and click `Enter`

- **Number of columns in the layout tables**

  Change 'Number of columns' to 2 and click `Enter`

- **Display results on the analysis page?**

  Tick check box by 'Tick box to display results on the analysis page' and click `Enter`

- **Process information**

  Ignore and click `Enter`

- **Graphical process information**

  Ignore and click `Enter`

- **Upload R script**

  Browse to location where you have saved the downloaded R script `dynmap_example.R` and click `Enter`

- **Upload subsidiary R scripts and data sources (optional)**

  In the top box type `dynmap_functions`

  Browse to location where you have saved the downloaded file `dynmap_functions.R` and click `Enter`

  In the top box type `meuse_grid`

  Browse to location where you have saved the downloaded file `meuse_grid.Rdata` and click `Enter`

  In the top box type `meuse_grid_ll`

  Browse to location where you have saved the downloaded file `meuse_grid_ll.Rdata` and click `Enter`

  Click `Finished entering subsidiary files`

- **Add an initial Login page?**

  Leave as default and click `Enter`

- **Which way to return results to user?**

  Leave as default and click `Enter`

- **Include a Cancel button on the web page**

  Ignore and click `Enter`

- **Create application**

  Click `Create application`

- **Completed**

  Click `dynmap_example.tgz` (or `dynmap_example.zip`, unpacked they are identical) to save file to your PC

- You are now finished with Rwui.

2.4 Installing Java

- Download the Java SE Runtime Environment (JRE) from:

  http://www.oracle.com/technetwork/java/javase/downloads/index.html

- Install the JRE on your PC according to the instructions included with the release, or linked to on the above page.

- Set an environment variable named `JRE_HOME` to the pathname of the directory into which you installed the JRE:

  On Windows if the path is e.g. `c:\jre6.0`, then

  – Right click on the My Computer icon on your desktop and select properties

  – Click the Advanced Tab

  – Click the Environment Variables button

  – Under System Variable, click New

  – Enter the variable **name** as JRE_HOME

  – Enter the variable **value** as the install path for the JRE e.g. `c:\jre6.0`

  – Click OK

  – Click Apply Changes

  Or on Linux if the path is e.g. `/usr/local/java/jre6.0` then

  – Add the line `export JRE_HOME=/usr/local/java/jre6.0` to file `.bash_profile`

  – Save

  – Run `source ~/.bash_profile`

- You may also use the full JDK rather than just the JRE. In this case set the `JAVA_HOME` environment variable to the pathname of the directory into which you installed the JDK, e.g. `c:\jdk6.0` or `/usr/local/java/jdk6.0`.

2.5 Installing Tomcat

- Download a binary distribution of Tomcat from:

  http://tomcat.apache.org

- Unpack the binary distribution into a convenient location on your PC.
- This will create a directory called eg. `apache-tomcat-6.0.35`.
- NB. On Windows - install into a directory called, for example, `C:\tomcat\apache-tomcat-6-0-35`. The path must not have any spaces, which prevent applications from running. Dots in the directory names also cause problems, as might other characters, so just use alphanumerics and hyphens as shown in the example.
- From here on we will refer to the path to Tomcat (eg `/usr/local/apache-tomcat-6.0.35` or `c:\tomcat\apache-tomcat-6-0-35`) as `TOMCAT_HOME`
- Tomcat can be started by executing the following commands:

  `\TOMCAT_HOME\bin\startup.bat` (Windows)

  `/TOMCAT_HOME/bin/startup.sh` (Linux)
- NB. On Linux make sure `startup.sh` is executable first
- Open a browser and type http://localhost:8080 in the address bar and this should take you to Tomcat's home page on your machine, indicating Tomcat is installed correctly and working.

2.6 Path to R

2.6.1 Linux

Nothing needs to be done on a Linux machine.

2.6.2 Windows

Need to set the path to R's bin directory

- Right click on the My Computer icon on your desktop and select properties
- Click the Advanced Tab
- Click the Environment Variables button
- In the 'System Variables' box scroll down and select variable 'Path' and press 'Edit'
- Add the path to R's bin directory by adding eg. `C:\Program Files\R\R-2.12.0\bin` to the ';' separated list
- Click OK
- Click Apply Changes

2.7 R package requirements

The R script requires the following packages to be available: sp, gstat, rgdal, raster.

R package rgdal requires two libraries installed:

- PROJ.4: Cartographic Projections Library http://trac.osgeo.org/proj/
- GDAL: Geospatial Data Abstraction Library http://www.gdal.org/

2.8 Installing the web application

- If you downloaded the tgz file unpack it: `tar zxvf dynmap_example.tgz`
- Or if you downloaded the zip file unpack it: `unzip dynmap_example.zip`
- A directory called `dynmap_example` will have been created by the unpacking
- Copy the file `dynmap_example/deploy/dynmap_example.war` to the directory:

  `/TOMCAT_HOME/webapps`
- Tomcat should unpack the `.war` file automatically, but if not simply stop and start Tomcat and the `.war` file should unpack.

  (Tomcat can be stopped by executing the following command:

  `\TOMCAT_HOME\bin\shutdown` (Windows)

  `/TOMCAT_HOME/bin/shutdown.sh` (Unix))
- This will give a directory `/TOMCAT_HOME/webapps/dynmap_example`

2.9 ProjectedOverlay.js

- Download file `ProjectedOverlay.js` from:

  http://code.google.com/p/geoxml3/source/browse/trunk/
- Copy file `ProjectedOverlay.js` to directory `/TOMCAT_HOME/webapps/dynmap_example/`

2.10 Sorting out the API key

- Follow the instructions for obtaining an Google Maps API key at:

  https://developers.google.com/maps/documentation/javascript/tutorial#api_key
- Then, in a text editor, open file `/TOMCAT_HOME/webapps/dynmap_example/EnterData.jsp`
- Locate the line (approx. line 31) reading:

  `src="http://maps.googleapis.com/maps/api/js?key=YOUR_API_KEY&sensor=false&libraries=geometry"`

- Replace `YOUR_API_KEY` with the api key you have just obtained from Google.
- (Note: if you don't currently have an API key you can simply remove the characters `key=YOUR_API_KEY&` from the above line)
- Do the same for file `/TOMCAT_HOME/webapps/dynmap_example/Results.jsp`

2.11 Using the web application

- First download a data file to analyse, eg. the zinc data file from:

  http://sysbio.mrc-bsu.cam.ac.uk/Rwui/tutorial/dynamic_map_tutorial/meuse_zinc.csv
- Open a browser and type `http://localhost:8080/dynmap_example` in the address bar.
- You should see your webapp, the same as http://sysbio.mrc-bsu.cam.ac.uk/dynmap_example
- Type `zinc` in the text box at the top
- In the File Upload box browse to location where you have saved the data file `meuse_zinc.csv`
- Select `ordinary` in the 'Display Kriging' drop-down list
- Click `Analyse`
- You should see the Google dynamic map with data points as color-coded circles and overlay of kriging predictions as shown in Figure 2.
- The opacity of the kriging predictions overlay can be varied by the controls below the map.
- The map can be zoomed and panned and the type changed to satellite imagery etc using the usual Google dynamic map controls.
- You will notice a link at the bottom of the page `Results for submission: ###########`, where `##########` is a string of characters. Clicking on this link opens a results page for this submission.
- Change the 'Display measurement points' to `bubbles`, the 'Display Kriging' selection to `universal`, and the 'Display prediction or standard errors' drop-down list to `se` and click `Analyse`.
- The display on the Google map will change according to the new selections and a further link to a results page will appear at the bottom of the page. The link to the first results page is still there too. In this way the results of previous submissions can still be accessed

2.12 Technical note

Note that the R script uses `png()` to create the overlay. `png()` allows you to create png files with transparent backgrounds which is just what you need for an overlay. `png` uses 'cairo', 'Xlib' or 'quartz' to create the png so R does need to be compiled with support for at least one of these in order to create the overlay successfully. And for `type = "Xlib"`, `png()` may not be usable unless the X11 display is available to the owner of the R process. `type = "cairo"` requires cairo 1.2 or later.

If png() is not supported as a temporary measure you could substitute the following code to create the overlay:

```
bitmap(file="overlay.png")
par(mai=c(0,0,0,0))
par(xpd=NA)
image(rotate.image(as.matrix(r.goog)), main="", bty="n", xaxt="n", yaxt="n", col=topo.colors(30))
dev2bitmap(file="overlay.png")
dev.off()
```

although the background will not be transparent. bitmap requires ‘ghostscript’ to be installed.

Figure 2: Screenshot of part of the web page of the Meuse soil pollution example application http://sysbio.mrc-bsu.cam.ac.uk/dynmap_example. Individual data points are marked as colour-coded circles and the results of kriging displayed as an overlay.

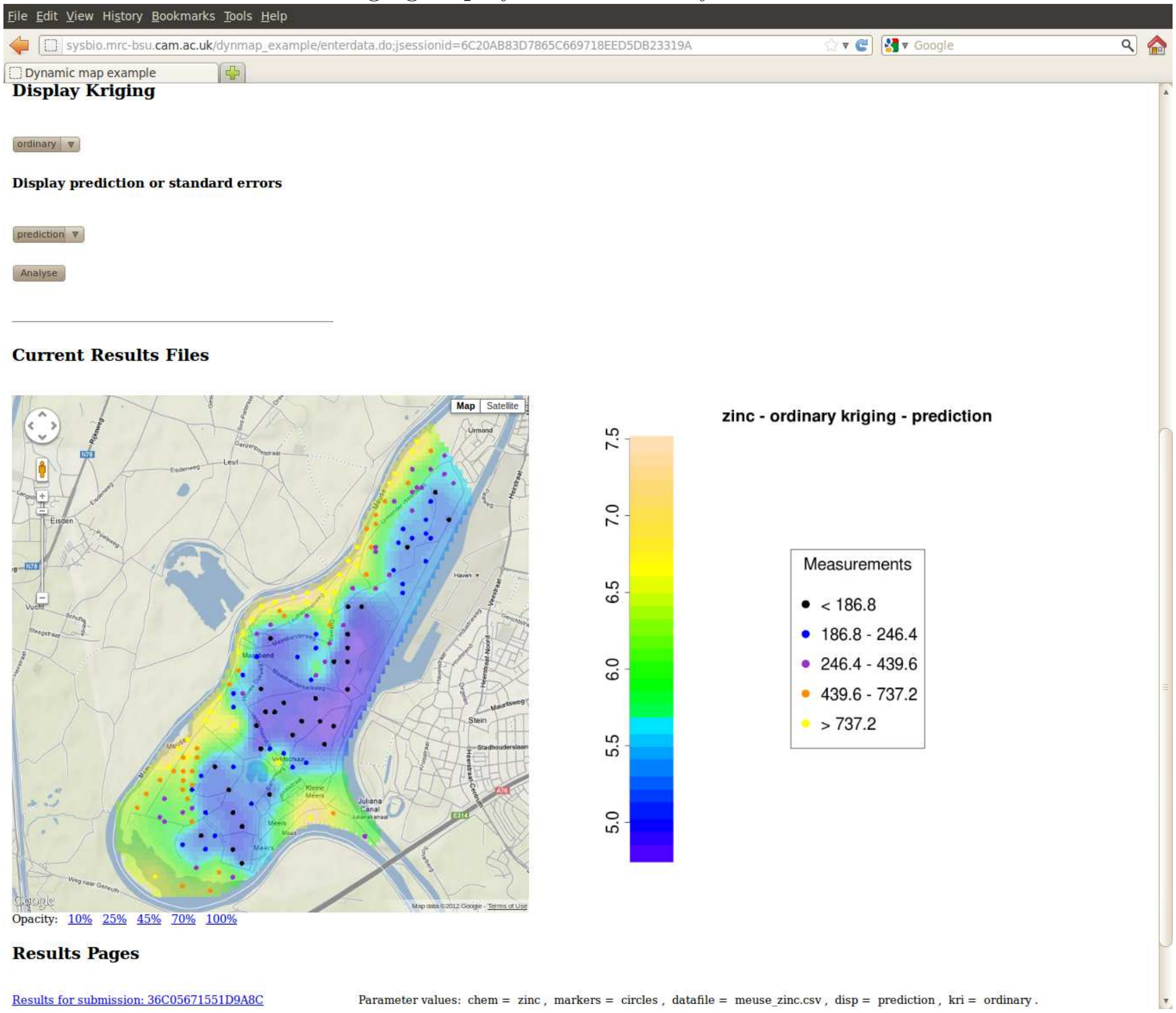

Supplement: Additional file 8 — Step-by-step guide to using Rwui to create webapps that display R script results on Google dynamic maps. [file 1476-072X-11-41-S8.pdf]
